# IBSEM: An Individual-Based Atlantic Salmon Population Model

**DOI:** 10.1371/journal.pone.0138444

**Published:** 2015-09-18

**Authors:** Marco Castellani, Mikko Heino, John Gilbey, Hitoshi Araki, Terje Svåsand, Kevin A. Glover

**Affiliations:** 1 Institute of Marine Research, P.O. Box 1870, Nordnes, N-5817, Bergen, Norway; 2 School of Mechanical Engineering, University of Birmingham, B15 2TT, Birmingham, United Kingdom; 3 Department of Biology, University of Bergen, Bergen, Norway; 4 International Institute for Applied Systems Analysis (IIASA), Laxenburg, Austria; 5 Marine Scotland Science, Freshwater Laboratory, Faskally, Pitlochry, PH16 5LB, Scotland, United Kingdom; 6 Research Faculty of Agriculture, Hokkaido University, Sapporo, 060–8589, Japan; Ecole normale superieure de Lyon, FRANCE

## Abstract

Ecology and genetics can influence the fate of individuals and populations in multiple ways. However, to date, few studies consider them when modelling the evolutionary trajectory of populations faced with admixture with non-local populations. For the Atlantic salmon, a model incorporating these elements is urgently needed because many populations are challenged with gene-flow from non-local and domesticated conspecifics. We developed an Individual-Based Salmon Eco-genetic Model (IBSEM) to simulate the demographic and population genetic change of an Atlantic salmon population through its entire life-cycle. Processes such as growth, mortality, and maturation are simulated through stochastic procedures, which take into account environmental variables as well as the genotype of the individuals. IBSEM is based upon detailed empirical data from salmon biology, and parameterized to reproduce the environmental conditions and the characteristics of a wild population inhabiting a Norwegian river. Simulations demonstrated that the model consistently and reliably reproduces the characteristics of the population. Moreover, in absence of farmed escapees, the modelled populations reach an evolutionary equilibrium that is similar to our definition of a ‘wild’ genotype. We assessed the sensitivity of the model in the face of assumptions made on the fitness differences between farm and wild salmon, and evaluated the role of straying as a buffering mechanism against the intrusion of farm genes into wild populations. These results demonstrate that IBSEM is able to capture the evolutionary forces shaping the life history of wild salmon and is therefore able to model the response of populations under environmental and genetic stressors.

## 1. Introduction

This paper introduces the Individual-Based Salmon Eco-genetic Model (IBSEM) and presents the results of its validation and calibration against empirical data from a well-described Atlantic salmon (*Salmo salar*) population. IBSEM was developed in response to a growing need to study and quantify evolutionary and demographic responses in native Atlantic salmon populations that are influenced by a range of anthropogenic challenges [1]. Among these challenges, genetic introgression from non-local fish, either released as part of a supplementary stocking campaign, or through domesticated farmed escapees, represents a long-standing issue that is the primary focus of the model’s application.

In several countries, marine cage-based salmon aquaculture represents a major industry. In Norway, which is the world′s largest producer of farmed Atlantic salmon, at any time, there are several hundred million farmed salmon in sea cages scattered along the coast (362 million salmon 31 December 2012 [2]). One of the major challenges with cage-based aquaculture in the marine environment is containment, and each year, thousands or hundreds of thousands of farmed salmon escape into the wild. Whilst most escapees disappear and do not return to freshwater to attempt to spawn [3][4], some mature and find their way to the spawning grounds of native populations, sometimes outnumbering wild conspecifics [5]. Whilst the spawning success of farmed escapees is known to be lower than wild salmon [6][7], domesticated farmed escapees have successfully interbred with wild salmon in some populations [8][9][10][11][12]. This is of major concern given that Atlantic salmon populations display considerable population genetic structuring [13][14] and life-history variation [15][16], and are therefore likely to be adapted to the rivers in which they originate [16][17]. Intrusion of non-native fish may disrupt these adaptations [18][19]. In addition, farmed salmon have been subject to artificial selection, and thus display a wide range of genetic differences to wild salmon. For example, when compared with wild salmon, farmed salmon display significantly increased growth rates [20][21][22][23] and reduced predator awareness [24], and, not least, the offspring of farmed escaped salmon display reduced survival in the natural environment [7][25][26][27]. Consequently, there are significant concerns regarding the potential long-term consequences that introgression of escaped farmed salmon have on recipient wild population´s viability and evolvability [28][29][30].

Understanding the potential impact of escapees on wild salmon populations requires quantitative knowledge on the biology of wild, farmed and hybrid salmon. Such knowledge is best synthesized using models.

Individual-based models allow designers to explicitly represent individual traits, which can be transmitted between generations [31]. They are particularly suitable to account for individual variability and life history, and hence genotypic and phenotypic diversity in populations. Individual-based models are most useful when intra-group diversity is important for understanding community and system dynamics [32]. Individual-based models need to cover the whole life cycle of the study species, and be sufficiently realistic in terms of the ecological forces acting upon the population and the description of inheritance of genetic material. Models that combine sufficient ecological and genetic realism can be termed as eco-genetic models [33]. Current individual-based models of salmon populations are either limited to the ecological representation [34][35], or lack detail for the genetic representation of the individuals [36].

IBSEM is a new eco-genetic model for Atlantic salmon populations, which simulates both the demographic and evolutionary dynamics of the modelled population. Differences in origin (wild, farmed, or hybrid) are reflected in the genotype of the individuals and affect the growth, survival, and maturation. IBSEM is parameterized in relation to empirical data from a single Norwegian river that has been studied extensively for many years. Importantly however, the range of output variables is within the values reported for other salmon rivers, and as such the model can be easily applied to many other salmon populations. The paper is structured as follows. Section 2 presents the IBSEM model, whilst section 3 describes the experimental validation. The results of the simulation tests carried out in section 3 are discussed in section 4. Section 5 concludes the paper.

## 2. The IBSEM Model

The life history of salmon [37] has been divided into three main phases: embryonic, juvenile and oceanic (adult). These phases broadly reflect the Atlantic salmon′s life history in the wild, and the timing of each phase has been set based upon knowledge of this life-cycle [38].

The embryonic phase conducted in freshwater is divided into two sub-phases: egg (egg) and alevin (al). It covers the period from the egg stage (egg) to emergence (al, first of April) and the first month of feeding. Fry is an intermediate stage between alevin and parr, and has been incorporated in the parr sub-phase in the present model. On the first of May, alevin enter the freshwater juvenile phase and become parr which feed and grow in the river. The juvenile phase is further divided into two broad sub-phases: parr and pre-smolt (henceforth just named smolt, sm). Parr are grouped according to age: young-of-the-year (p0), one year old (p1), and older (p2) individuals. Individuals in the parr sub-phase grow for a variable number of years, until they enter the smolt sub-phase. The probability of smolting depends on the size attained by the individual. A year at the parr sub-phase is divided in a growth season (warmer months, March–October) and a resting season (colder months, November–February) where growth halts and mortality decreases. Male parr may mature in October, and take part to the reproduction process at the end of the month. Reflecting the costs of gonad development, maturing parr do not grow in size during the month of October.

Juvenile salmon remain six months (November–April) in the smolt sub-phase, and enter the adult phase and migrate to sea on the first of May. Smolts grow also in the resting season when water temperatures are very low, albeit at a reduced rate.

Within the model, the adult marine phase lasts up to three years, until the individual returns to the river to complete the life-cycle and spawn. For simplicity, all adult salmon are set to return to freshwater in October, whilst the probability of returning depends on the age and genotype of the individual. A fraction of the adult spawners survive after reproduction (known as kelts) and re-join the adult population in the sea. In the adult phase there is no resting season, and growth and mortality are calculated according to the same parameters all year. Adult salmon are grouped according to sea age, which is measured as the number of full winters spent at sea: first year at sea (0SW), one year (1SW), two year (2SW), and three or more years (3SW) old.

**Fig 1:**
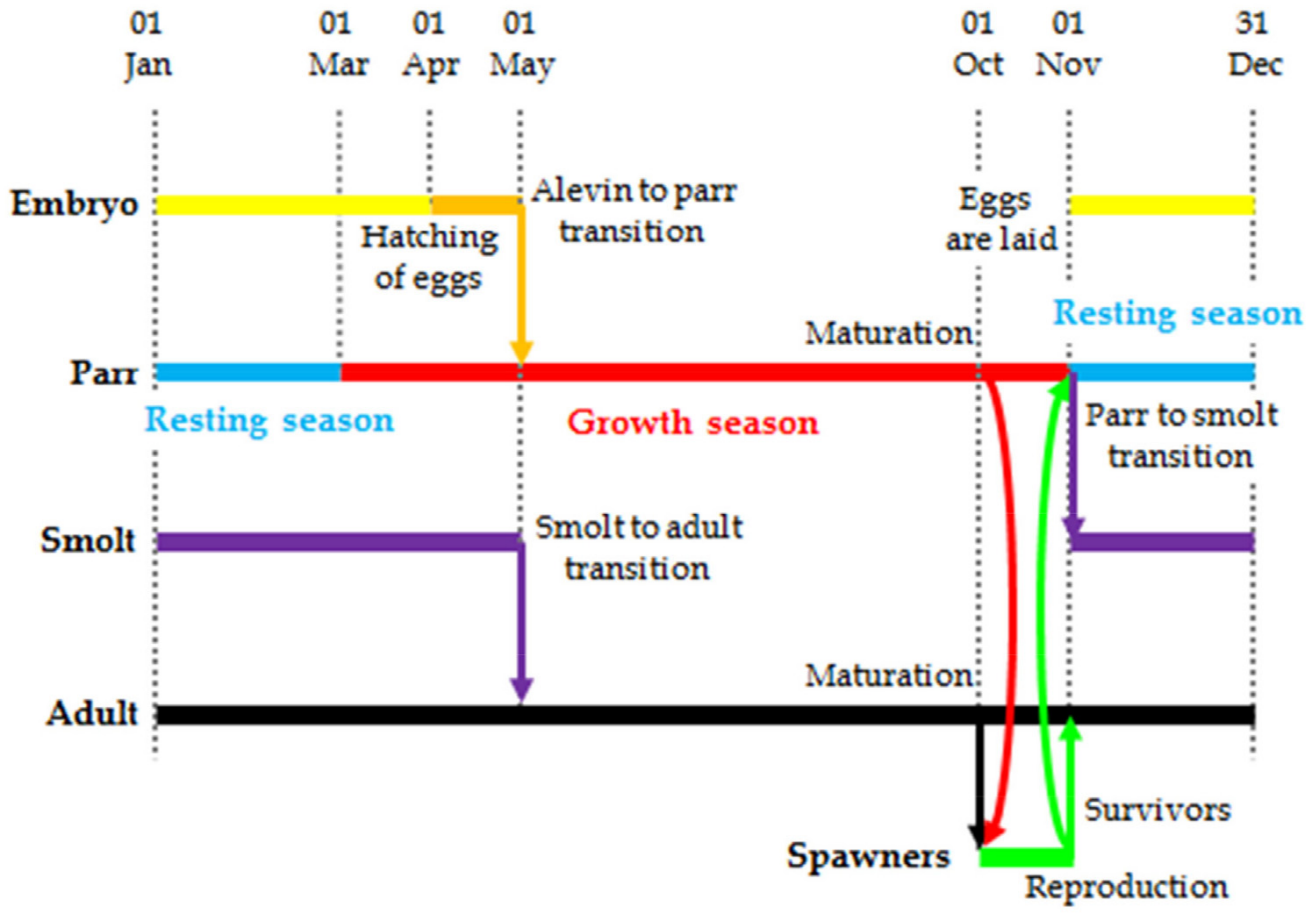
Life history of modelled salmon population

The main events in the life history of the individuals are summarised in Fig 1.

The model has been tuned to reproduce the life cycle of salmon in the river Os in Hordaland, western Norway (60°N, 5°E). The river Os was chosen because a long and detailed time series covering 25 years in juvenile and adult population measurements, and environmental variables, have been reported, enabling realistic parameterization [39]. However, the observed values from the model fall well within the range of values observed for other salmon populations [40]. The model can be easily re-parameterized for other populations.

The remaining part of this section describes the principles and parameterization of the model. A detailed description of the model equations is given in S1 File, whilst the parameter settings are given in S2 File.

### 2.1 Physical Environment

The physical environment is described using two variables: the river area and the monthly average of the water temperature. They are modelled on those encountered in the Os river [39] (Table A in S2 File). For the oceanic phase, the water temperatures are set to simulate the monthly average sea surface temperatures (SSTs) measured in the period 1982–2012 in the Norwegian Sea (NOAA_ERSST_V3 data provided by the NOAA/OAR/ESRL PSD, Boulder, Colorado, USA, http://www.esrl.noaa.gov/psd/). For each month, the water temperature can be fixed for all years of the simulation at the recorded average value, or randomised according to a Gaussian distribution centred on the average and with the standard deviation set to mimic the fluctuations of temperatures in the river Os and in the Norwegian Sea.

### 2.2 Inheritance

A genetic component is built into the model, permitting the tracking of genotypic and phenotypic distributions over time. The genes underlying documented genetic differences in growth, survival and maturation between wild and farmed domesticated Atlantic salmon, however, remain still largely unknown, despite the gene transcription [41][42] and linkage mapping studies [43] that have attempted to identify them. Furthermore, a range of genetic studies investigating neutral or close to neutral genetic markers such as microsatellites and single nucleotide polymorphisms have observed allele frequency differences between strains of farmed and wild salmon populations [44][45][46]. However, these allele frequencies are strongly population specific (i.e., vary also among wild populations and among farmed populations), and do not represent the differences for fitness-related genes behind the divergence between farmed and wild salmon. Therefore, in order to produce a set of genes that permit coding the functional differences between farm and wild salmon, the following system has been implemented in IBSEM.

Empirical observations have suggested that a significant proportion of the genetic basis for observed growth [21][22][23] and fitness [26] differences between wild and farmed salmon can be explained by an additive model. McGinnity et al. [26] found significant differences in the lifetime reproductive success of wild, hybrid and farmed fish. Fitting a simple additive model of effects, based on the proportion of wild or farmed origin alleles possessed by the different hybrids, to the lifetime reproductive success estimates obtained in their study (i.e. proportion of wild type alleles vs. reproductive success; see their Table 1), McGinnity et al. [26] found that the simple additive proportion of wild type alleles explained 87% of the fitness differences observed. In the absence of information as to the other components of genetic architecture (dominance, epistatic, and pleiotropic effects) that may be influencing the traits under investigation, we have chosen to use an additive model here.

**Table 1:** Test scenarios, initialisation of demographic parameters.

Parameter	Value
Starting month	May
Size of initial parr0 population	600000 (2.4 fish/m^2^)
Size of initial parr1 population	30000 (0.12 fish/m^2^)
Size of initial parr2 population	30000 (0.12 fish/m^2^)
Size of initial smolt population	0 (0 fish/m^2^)
Size of initial adult population	1000
Average fork length (mm) at parr0 sub-phase.	23
Average fork length (mm) of parr1 sub-phase.	65
Average fork length (mm) of parr2 sub-phase.	100
Average fork length (mm) of adults (0SW, 1SW, 2SW, 3SW).	140, 560, 670, 800
Average frequency of wild ‘1’ allele in population	0.9 (0.1 in scenario 2)

Within IBSEM, the distribution of genetic effects across a set of 21 loci is modelled via an exponentially declining effect distribution (see Fig five in [47], and Fig A in S1 File). Accordingly, traits are mostly influenced by a few genes, whilst the remaining genes have exponentially smaller effect. Evidence for this declining model of gene influences has been seen in both livestock (see meta-analysis in [48]) and salmon [49]. Such a distribution is also consistent with evolutionary theory and the fixation of alleles of large effect first under selective pressures [47], which are particularly strong in aquaculture selection programmes. For IBSEM, the last locus (21) is set to have no phenotypic effect (i.e., represents a completely neutral marker) and can therefore be used to track genetic introgression in the population.

The influence of the genetic architecture on the fish in the different phases of the life history is modelled using three separate sets of 21 genes, one set for each of the three main life phases: embryonic (egg to the end of endogenous feeding on its embryonic yolk-sac reserves), freshwater/juvenile, and marine/adult. The differences in life-history characteristics in these three phases are significant. It is thus reasonable to assume that different sets of genes influence the traits in these environments. Such a hypothesis is also strengthened by empirical evidence in a number of model organisms which suggest ontogenetic changes in groups of loci (or operons) influencing phenotypic traits at different life-history phases [50][51][52][53][54][55]. Similar evidence of ontogenetic changes in operon expression has also been seen with salmonids [34]. Since the model is aimed at the simulation of contemporary evolution, mutations are not included.

IBSEM uses a binary representation where each locus can take up either a ‘0’ or a ‘1’ allele. Genetic differences amongst farm and wild fish are encoded through different allele frequencies. Individuals of wild origin are initialised with frequency 0.9 of wild ‘1’ alleles, whilst individuals of farm origin are initialised with frequency 0.9 of ‘0’ alleles. Therefore, allele frequencies near the extremes of the range 0–1 characterise ‘pure’ farm and wild salmon, respectively, whilst intermediate frequencies denote hybrids. Since standard wild (farm) genotypes include ‘0’ (‘1’) alleles at low frequencies, a certain genetic diversity within the types is allowed. It is important to notice that our scheme aims at representing only those loci that diversify farm from wild individuals.

The choice of representing wild and farm salmon with respectively 0.9 and 0.1 frequency of ‘1’ alleles is motivated by convenience. Different parameterizations of the frequencies could have been used (e.g. 0.8 and 0.2 for wild and farm fish respectively). As long as we use an additive linear model of genetic effects, the effect of using different frequencies would have been equivalent to the effect of modifying the phenotypic differences between wild and farm fish (see Fig A.2 in S1 File).

In summary, the IBSEM genetic component is built upon the following characteristics:

- Reproduction is sexual. The sex of the individual is randomly determined at birth, with an equal sex ratio.
- Individuals are diploid (two sets of chromosomes) and inherit a maternal and a paternal allele per locus at random.
- Loci are independent.
- Binary representation of allelic values.
- Genetic effects on traits follow an additive linear model.
- The distribution of genetic effects across a given set of loci is exponentially declining, with the 21^st^ locus being completely neutral.
- The genotype is composed of three independent sets of genes, each defining the individual traits in one of the three main life phases (embryonic, juvenile, adult).
- The final phenotypic value (or trait value) of an individual includes a random factor which represents developmental and environmental noise in phenotypic expression. A detailed description of the genetic component of the model is given in section A.3 in S1 File.

### 2.3 Demography

Each individual is defined by a number of attributes, namely genes, sex, life phase, freshwater and sea age, length, phenotypic weight (final weight determined by genotypic weight and environment), and sexual maturity. Individuals of all life phases are subject to growth and mortality. Once they have attained the threshold size, juveniles (parr) undergo smolting, which marks the transition from the freshwater juvenile to the oceanic adult phase. Finally, individuals mature sexually (either as precocious male parr, or after the oceanic phase) and participate in reproduction.

#### 2.3.1 Growth

Somatic growth depends on an individual’s current size, genotype, age, and life phase, as well as on environmental variability (modelled as additive noise), and the following environmental variables: season, water temperature, and fish density. In the embryonic phase, the final alevin fork length is strongly correlated with egg diameter [34][56]. A random component is added to model environmental and developmental noise, with the aim to replicate the distribution of alevin length modelled by Gilbey and Verspoor [34].

In the juvenile and adult phases growth is affected by age, genotype, water temperature, and environmental stochasticity. The potential for growth is assumed to be highest in the first year of each phase, and gradually decreases in subsequent years. At the parr sub-phase, growth is also adversely affected by population density (competition for food) and is limited to the warm season (March–October). In the remaining months, parr are assumed to go into a resting state.

According to the predominant literature, growth is modelled according to an allometric relationship between growth rate and fish mass, and occurs only within a given interval of water temperatures (Eq. 3 in [57]; Eq. 10 in [58]; Eq. 14 in [36]). Juvenile and adult farmed salmon are set to grow faster than wild salmon. The genotypic effect on growth is adjusted heuristically to simulate observed differences between wild and farmed individuals [20][21][22][23][27], and will be the object of a sensitivity analysis in section 3.3.

At the parr sub-phase growth is density-dependent. Density effects on parr growth depend on the effective density ED [36][59] of the population. In the smolt and adult phases, growth is density-independent.

Parr and smolt growth equations are parameterized based on literature [57][58], adjusting the values heuristically to reproduce, at comparable population density and age, the average lengths observed in the river Os salmon [39]. Environmental variability has been modelled via an additive random component tuned to replicate the distribution of fish weights observed in the salmon population from the river Os.

Growth at the adult phase was tuned to obtain comparable averages and standard deviations on the size of returners as those measured by Jonsson et al. [40] in a range of salmon rivers. A detailed description of the growth equations is given in section A.4.1 in S1 File, whilst the parameters are given in section B.2.1 in S2 File.

#### 2.3.2 Mortality

According to the life phase, mortality depends on environmental variables including season, fish density, and randomness, and individual state variables including genotype and size. Salmon of wild origin are given higher survival probabilities than salmon of farm origin. Since there is no consensus in the literature over the differential in mortality between wild and farmed salmon [7][26][27], the effect of different parameterizations will be the subject of a sensitivity analysis.

In each life phase, the effects of all the factors contributing to mortality are compounded into an overall probability m (Eqs. A.19, A.22-A.24 in S1 File). For each individual, a random number r in the interval [0,1] is drawn, and if r<m the individual dies.

In the embryonic phase, egg-to-alevin mortality depends on genes, egg size, and density. The effect of egg density (egg/m^2^) on embryo mortality is calculated to fit the exponentially increasing curve used by Gilbey and Verspoor [34]. Data from the Guddalselva river in Hordaland, Norway [27] suggest a positive correlation between egg size and egg-to-alevin survival in the first weeks after first feeding. Einum and Fleming [60] found a similar positive relationship (although with a different functional form), as did Solberg et al. [61] in a semi-natural environment. In IBSEM, egg-to-alevin survival probabilities are linearly related to egg size.

The calculation of mortality probabilities in the juvenile phase is based on Piou’s and Prevost’s estimate of daily survival probabilities [36], adjusted to obtain densities resembling those found in the river Os [39]. Mortality probability decreases with the freshwater age of the individual [36], and are higher in the warm season when the fish are actively feeding [36]. During the cold season, individuals are assumed to be at rest and mortality is lower. Juvenile salmon are considered to be feeding throughout the whole smolt sub-phase, and survival probabilities are kept the same in the cold (November–February) and warm (March–April) seasons. Smolts have higher survival than parr of equal age during the warm season [36], and higher (p0) or equal (p1, p2) to those of same aged parr during the cold season.

Density effects are considered significant at the parr sub-phase, but negligible at the smolt sub-phase and adult phase. They are calculated using the effective fish density of the population (Eq. 13 in [59]). Marine survival is related to individual size and genotype. Sea mortality rates are taken from Piou and Prevost [36], slightly modified to obtain realistic return rates in the river Os [39].

A detailed description of the mortality equations is given in section A.4.2 in S1 File, whilst the parameters are given in section B.2.2 in S2 File.

#### 2.3.3 Maturation

Male Atlantic salmon can mature at sea or in freshwater as parr. In male parr, Piche et al. [62] reported variation in the incidence of maturity, as well as in the reaction norms between maturation and growth rate. Myers et al. [63] and Whalen and Parrish [64] postulated the existence of size thresholds for maturation, although their estimates for such thresholds are age-specific (respectively 70 and 100mm in June–August for maturation of p1 and p2 in November). Evidence of genetic variation influencing parr maturation within populations is still inconclusive (but see Skilbrei and Heino [65]). We took a conservative approach and related the maturation probability only to the length of the individual.

In the model, the maturation probability (see Fig A in S2 File) is a sigmoidal function of length [34]. Based on empirical observations at the Institute of Marine Research—Bergen (IMR), male parr that matured the previous season are given a maturation probability two times larger (capped to one) than the probability given to parr that have not previously matured. Gonad development is assumed to take up resources. For this reason, maturing parr do not grow the last month (30 days) of the warm season.

In the river Os, 43, 93, and 100% of respectively one year, two year, and older male parr were found sexually mature during a survey carried out in October 2010 [39]. Maturation probabilities are set to obtain similar percentages of maturity for male parr of similar size.

In the model, adult salmon spend between one and three winters at sea before they return to spawn. Maturation is related to genotype and randomness: wild fish have higher probabilities to return earlier to spawn than farmed salmon. At each sea age, individuals have a fixed, age-dependent maturation probability.

Maturation probabilities in adults take into account the observations of Kallio-Nyberg and Koljonen [66], the parameters used by Hedger et al. [35], and local expertise at the IMR. Farmed salmon have been genetically selected for delayed age of maturation [67][68]. In nature, farmed salmon typically return as multi-sea winter fish [25][26], whilst fish in the river Os typically return as 1SW, 2SW and occasionally 3SW old [39]. The differential in probabilities between pure wild and farmed adult salmon was set taking into account these differences. A detailed description of the maturation equations is given in section A.4.3 in S1 File, whilst the parameters are given in section B.2.3 in S2 File.

#### 2.3.4 Smoltification

Pre-smolts begin to differentiate in size from other parr of the same age towards the end of October, and by the time of the migration to sea, the population of parr and pre-smolts is characterised by bimodal length distribution representing these two components of a cohort. The ‘decision’ on whether to maintain sustained growth in winter or enter a rest state until next spring is dependent on the size attained by the individual at the end of the summer [69][70][71][72].

Many authors [71][72][73] have suggested the existence of a minimum length threshold of the pre-smolt sub-phase. Threshold lengths required to enter the pre-smolt stage seem to vary amongst populations of Atlantic salmon [74]. For Canadian populations, Kristinsson et al. [72] suggested a size threshold of 80–120 mm at the end of the summer, whilst Skilbrei [74] found that Norwegian populations begin to separate in two growth modes at smaller size (70–80 mm). In their survey in the river Os, Rådgivende Biologer AS [39] found no pre-smolts smaller than 100 mm. We have set conservatively the threshold to enter the smolt sub-phase to 90 mm.

The smolting probabilities of individuals longer than the 90 mm threshold are calculated using a logistic function of size (Fig A in S2 File). The function was parameterized in order to fit the size distribution of pre-smolts found in the river Os by Rådgivende Biologer AS [39] in October 2010. The parameterization gives a 50% smolting probability for parr of 103 mm length. Piou and Prevost [36] and Hedger et al. [35] parameterized similar logistic equations using data respectively from the river Scorff in France, and the river Stryn in Norway. They obtained respectively 89 and 113 mm as the size at 50% smolting probability.

The probability of smolting the following May is calculated at the end of October for each individual. A detailed description of the smolting equations is given in section A.4.4 in S1 File, whilst the parameters are given in section B.2.3 in S2 File.

#### 2.3.5 Reproduction

In the model, reproduction takes place at the end of October and involves sexually mature male parr and adult males and females.

In nature, mature male parr are often attacked by large adult males on the spawning sites, represented here as pre-spawning mortality. Within the model, the mortality probability for the population of mature male parr is randomly sampled from a pre-set range of values each year. After spawning, adult salmon have a fixed mortality probability. Angling is a major source of adult mortality in wild salmon populations where this is permitted, but this is not included in the model at present. Parr pre-spawning and adult post-spawning mortality probabilities are set according to local expertise at the IMR.

A spawner’s body mass influences fecundity and egg size [36][56][75]. In the model, we calculated the average fecundity and egg weight for each individual using the linear relationships estimated by Jonsson et al. [75]. We obtained the actual numbers and weight of eggs by randomly sampling Gaussian distributions centred on these means, with the standard deviations chosen to mimic the spreads used by Gilbey and Verspoor [34].

Each female is paired with up to 2 mature adults and 5 sexually mature parr. The percentage of eggs fertilised by parr is randomly determined as in Gilbey and Verspoor [34], using a Gaussian distribution of probabilities defined by a mean of 30% and a standard deviation of 10%. Within the two pools of adult and juvenile males, fertilisation opportunities are allocated according to individual size (weight). Farmed escapees are given a lower spawning success than fish of any genetic make-up that are born in the wild [6]. A detailed description of the reproduction equations is given in section A.4.5 in S1 File, whilst the parameters are given in section B.2.4 in S2 File.

#### 2.3.6 Straying

Not all wild salmon return to spawn in their natal river [76][77]. Fish that stray between rivers allow established populations to colonize new habitats, and counteract possible inbreeding effects.

In the model, straying is used to support the genetic diversity of the focal population. Every year, a fixed fraction of the adult returners is randomly picked and removed from the population (thus representing fish that stray into neighbouring rivers). Each of these strayers is replaced with a newly generated “stray” individual. To take into account lack of local adaptation to the river, incoming strayers are characterised by a frequency (0.8) of wild alleles, slightly lower that the frequency in wild salmon (0.9, see Section 2.3). No assumption can be made on the phenotypic attributes of the incoming strayer. For simplicity, they are set to replicate those of the outgoing strayer.

The fraction of strayers is heuristically set to 5% of the returners, which is within the range of values reported in empirical studies of straying in Atlantic salmon [78][79][80]. A detailed description of the straying equations is given in section A.4.6 in S1 File, whilst the parameters are given in section B.2.4 in S2 File.

## 3. Simulation Tests and Model Validation

The performance of the IBSEM model is tested in five simulation scenarios. The purpose of the first scenario is to prove that the model is able to reproduce the salmon population dynamics of a natural river. The purpose of the second scenario is to verify whether the emergent population reaches an evolutionary equilibrium that is close to our definition of a ‘wild’ genotype. The purpose of the last three scenarios is to show the sensitivity of the results to the assumptions made on the differences between salmon of wild and farm origin.

In scenario 3, the differences in growth and mortality between farmed and wild salmon are set to their ‘standard’ values (Section B.3 in S2 File). In the last two scenarios, the differences in growth and mortality between farmed and wild salmon are modified. In the first case, the differences are reduced to 50% of the original value (Section B.3 in S2 File), in the second case they were increased by 50%. The remainder of this section details the tests and experimental results.

### 3.1 Scenario 1 –Reproduction of the Population Dynamics of the River Os

The purpose of the first test is to ascertain how closely the IBSEM model reproduces the population dynamics in the river Os in accordance with a long time-series of measurements in this river [39]. The performance of the model is evaluated on the results of ten independent runs (replicates). For each run, the salmon population is let to evolve for 100 years (settling period), and then monitored for additional 100 years (monitoring period). We found experimentally that a settling period of 100 years guarantees that the simulated salmon population has set into a stationary state.

In the monitoring period, the initial egg population is sampled after its creation at the end of October. The parr population is sampled twice a year: on the first of May (beginning of growth season for young-of-the-year) and end of October (end of growth season). The smolts are sampled on the first of November (at the time smolts start differentiating from parr) and at the end April (prior to seaward migration). Oceanic salmon are sampled once a year at the end of September (before returners migrate back to freshwater). The pool of spawners is sampled at the time of its formation at the beginning of October (see Section 2). For each sample, the main demographic parameters including the density or number of individuals, average and standard deviation of the length or weight of the individuals, and mortality rate, are recorded.

For the density and mortality data as well as length and weight of the individuals, the arithmetic mean and standard deviation are calculated over the 100 monitoring years, and averaged over the 10 replicates. For all records, the minimum and maximum values over the 100 years and 10 replicates are also calculated. Throughout the tests, we found out that 10 replicates allowed us to capture the variability of the model, whilst keeping overall running times acceptable. Where possible, the results are compared with available data from the river Os [39] or other salmon rivers where there is available data (e.g. [40]).

#### 3.1.1 Scenario 1 –Model initialization

IBSEM is parameterized as in Sections B.1 and B.2 in S2 File (default values). The initial values of the population parameters are detailed in Table 1. Generally, these values reflect values typical for the salmon population in the river Os [39]. The simulation starts in May after alevin turned into parr, and smolts migrated to sea. The initial population includes parr (young-of-the-year, p1 and p2) and adults (uniformly distributed amongst 0SW, 1SW, 2SW, and 3SW ages).

#### 3.1.2 Scenario 1 –emergent population sizes and densities

**Fig 2:**
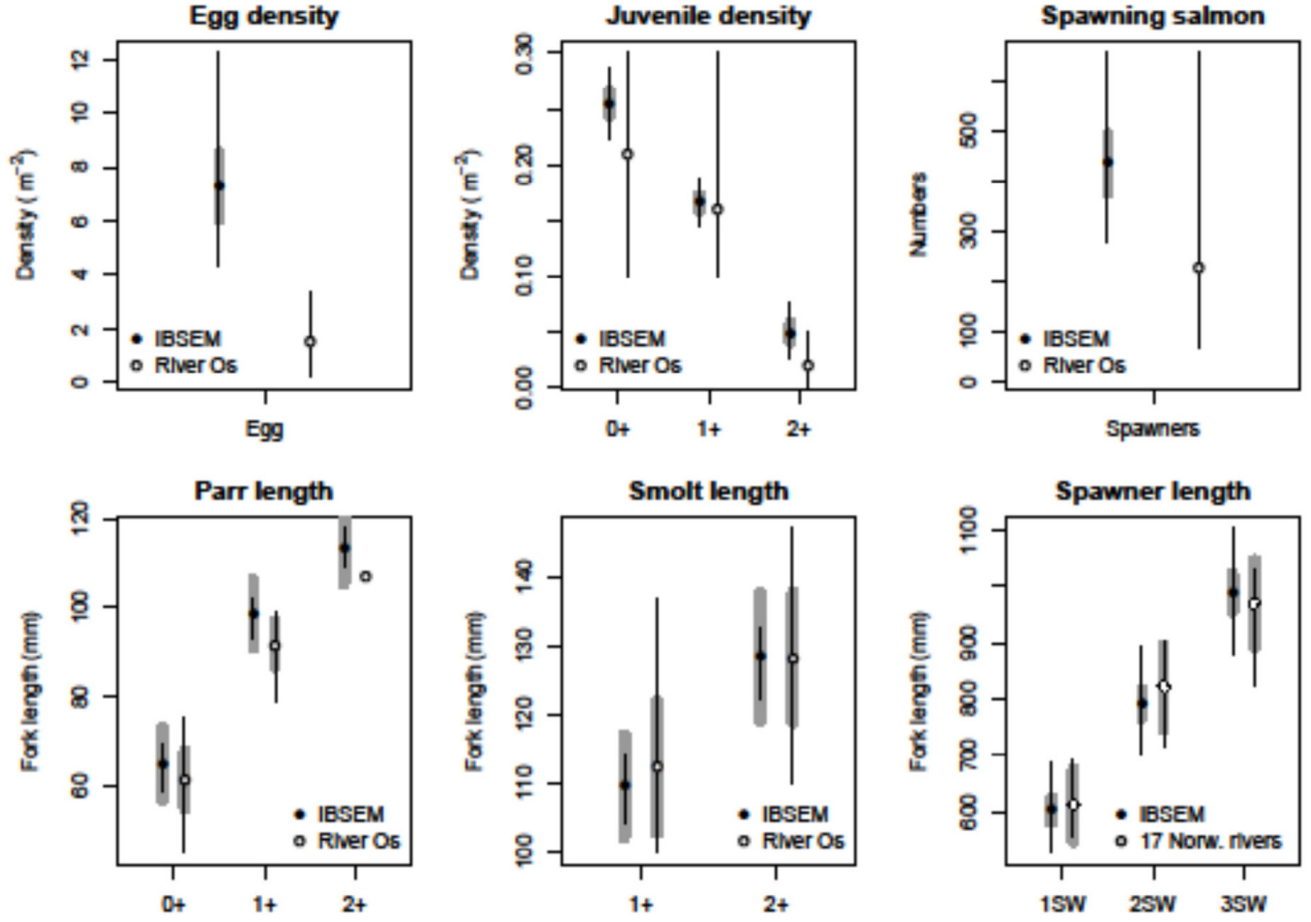
Scenario 1—Demographic data of the emergent salmon population. Figures are broken down per life phases, and report population averages and standard deviations (thick grey lines) over 100 years, averaged over 10 independent runs of the model. The overall minimum and maximum values over the 100 years and 10 independent runs are also reported (whiskers). The results are compared with observations in the Os river (Rådgivende Biologer, 2012) or other biological populations (N. Johnson et al. 1991).

The main demographic data of the emergent salmon population are compared with those of the population in the river Os or other Norwegian rivers in Fig 2. The complete set of demographic results broken down for life phases and year classes is given in Tables 2–7. Generally, the structure of the simulated population is characterised by reasonably low standard deviations and ranges, and the model generates consistent and repeatable demographics. The average densities and population sizes at various life phases remain constant (not shown) throughout the 100 year monitoring period, confirming that the system has reached a stationary state characterised by a relatively stable population undergoing moderate stochastic fluctuations.

**Table 2:** Scenario 1—Demographics of oceanic salmon population.

Date	31-September				
Source	Model				
Age	0SW	1SW	2SW	3SW	Total
**Average**	1338	540	211	29	2118
**Stdev**	214	98	42	7	239
**Min**	696	271	104	10	1405
**Max**	2148	960	373	59	3040

Population size information is broken down per age groups. The table reports the averages and standard deviations over 100 years, averaged over 10 independent runs of the model. The overall minimum and maximum values over the 100 years and 10 independent runs are also reported.

**Table 3:** Scenario 1—Demographics of spawners population.

Date	01-October				Mid-October (1992–2008)
Source	Model				Os river
Age	1SW	2SW	3SW	Total	Total
**Average**	223	184	29	436	228
**Stdev**	42	38	8	58	-
**Min**	112	87	10	280	67
**Max**	437	324	60	656	656

Population size information is broken down per age groups. The table reports the averages and standard deviations over 100 years, averaged over 10 independent runs of the model. The overall minimum and maximum values over the 100 years and 10 independent runs are also reported. The results are compared with statistical measures of population surveys carried out in the Os river in the period 1992–2008 (Rådgivende Biologer AS, 2012).

**Table 4:** Scenario 1 –Egg density.

Source	Model	Os river (1991–2010)
**Average**	7.298	1.5
**Stdev**	1.238	-
**Min**	4.317	0.2
**Max**	12.260	3.4

Statistical measures of simulated egg densities per m^2^. The tables report the averages and standard deviations over 100 years, averaged over 10 independent runs of the model. The overall minimum and maximum values over the 100 years and 10 independent runs are also reported. The results are compared with averages of estimated egg densities in the Os river in the 1989–2010 period (Rådgivende Biologer AS, 2012).

**Table 5:** Scenario 1—Demographics of parr population.

Date	01-May				01-November			
Source	Model				Model			
Age	P0	P1	P2	Total	P0	P1	P2	Total
**Average**	2.4257	0.2094	0.0655	2.7006	0.2549	0.0746	0.0016	0.3311
**Stdev**	0.2971	0.0087	0.0117	0.2888	0.0103	0.0132	0.0008	0.0186
**Min**	1.7377	0.1827	0.0369	2.2749	0.2229	0.0424	0.0004	0.3033
**Max**	3.5186	0.2360	0.1089	3.1913	0.2879	0.1224	0.0069	0.3616

Statistical measures of parr densities per m^2^ in May (alevin emergence) and at the end of the growth season. Density data are broken down per life phases and age groups. The tables report the averages and standard deviations over 100 years, averaged over 10 independent runs of the model. The overall minimum and maximum values over the 100 years and 10 independent runs are also reported.

**Table 6:** Scenario 1—Demographics of smolt population.

Date	01-November				31-April			
Source	Model				Model			
Age	Smolt0+	Smolt1+	Smolt2+	Total	Smolt0+	Smolt1+	Smolt2+	Total
**Average**	0.0000	0.0910	0.0480	0.1390	0.0000	0.0755	0.0402	0.1157
**Stdev**	0.0000	0.0117	0.0084	0.0117	0.0000	0.0098	0.0070	0.0098
**Min**	0.0000	0.0481	0.0261	0.1038	0.0000	0.0398	0.0219	0.0862
**Max**	0.0002	0.1198	0.0749	0.1793	0.0001	0.0999	0.0623	0.1496

Statistical measures of smolt densities per m^2^ at the beginning and end of the smolt sub-phase. Density data are broken down per life phases and age groups. The tables report the averages and standard deviations over 100 years, averaged over 10 independent runs of the model. The overall minimum and maximum values over the 100 years and 10 independent runs are also reported.

**Table 7:** Scenario 1—Demographics of juvenile population.

Date	01-November				Mid-October (1991–2010)		
Source	Model				Os river		
Age	0+	1+	2+	Total	0+	1+	2+
**Average**	0.2549	0.1656	0.0496	0.4701	0.21	0.16	0.02
**Stdev**	0.0103	0.0070	0.0082	0.0184	-	-	-
**Min**	0.2229	0.1444	0.0277	0.4159	~ 0.10	~ 0.10	~ 0.00
**Max**	0.2879	0.1882	0.0765	0.5287	~ 0.30	~ 0.30	~ 0.05

Statistical measures of juvenile (parr+smolt) densities per m^2^ at the beginning of the resting season. Density data are broken down per life phases and age groups. The tables report the averages and standard deviations over 100 years, averaged over 10 independent runs of the model. The overall minimum and maximum values over the 100 years and 10 independent runs are also reported. The results are compared with statistical measures of population surveys carried out in the Os river in the period 1991–2010 (Rådgivende Biologer AS, 2012).

In the simulations (Fig 2), egg densities range above 7 eggs/m^2^, with an overall minimum and maximum of respectively 4.3 and 12.6 eggs/m^2^. The average standard deviation is relatively narrow. Egg densities in the river Os were estimated to range widely (0.2–5.5 eggs/m^2^) in the 1989–2011 period [39]. These Figs only include eggs of wild salmon origin, and thus underestimate the total density. Different studies put the optimal egg density target for the river Os between 3 and 6 eggs/m^2^ [39]. In general, the model settles on a regime of average egg densities that is somewhat higher than the average in the river Os. However, given that the smolt production curve is known to flatten at egg densities above the optimal [81][82], it is plausible that the average figure obtained in the simulations is compatible with near-optimal smolt production. The possibility that the real egg density in the river Os (calculated from the number of returners) might have been underestimated should also be considered.

In the simulations, the density of juvenile salmon (parr + smolt) on 01-November (when smolts start differentiating in size from parr) is 0.5 fish/m^2^, similar to the mid-October estimate of 0.56 individuals/m^2^ in the river Os [39]. A direct comparison between the experimental and simulated juvenile densities per year class is not straightforward. The results of a survey are highly dependent on the environmental conditions (water flow and temperature) in the immediately preceding years. In the model, only changing temperature is modelled, which may reduce yearly population variability. Comparison with the results of the 1991–2010 surveys shows that the model captures the population dynamics, and gives realistic densities for the three age classes (Fig 2).

The average smolt density in the river Os on 15-10-2010 was 0.25 fish/m^2^ [39], and in the 20 years between 1991 and 2010 was 0.17 fish/m^2^ (range 0.1–0.3). The simulated smolt densities at the end of October (Table 6) are in good agreement with those in the river. Smolt production in the river Os is estimated to 0.15 fish/m^2^ [39], similar to the 0.12 smolts/m^2^ obtained by the model (Table 6), and in line with the range of values (0.003–0.3) found by Jonsson et al. [83] in the river Imsa.

The average number of returners in the river Os in the period 1992–2008 ranged from 67 to 656, with an average of 228 adults per year. The average number of adult spawners in the model is slightly higher than in the river, but well within the observed range (Fig 2).

Overall, the model gives moderately higher egg and adult densities than those observed in the river Os. However, current population densities in the river might be below optimal values due to increased marine mortality due to salmon louse infection (*Lepeophtheirus salmonis*) [84], and overharvest through angling [39]. Indeed, egg densities in the 1989–2010 period have been consistently below the recommended targets for sustainability [39]. The effects of sea lice infestation and overfishing are not factored into the model.

#### 3.1.3 Scenario 1 –emergent population, size of individuals

The distribution of individual fish sizes in the emergent population at different life phases are compared with empirical data from the river Os or other Norwegian rivers in Fig 2. The experimental results are detailed for different life phases and age classes at different times of the year in Tables 8–11.

**Table 8:** Scenario 1 –Average parr fork length.

Date	01-May			01-November			15-October		
Source	Model						Os river		
Age	P0	P1	P2	P0	P1	P2	P0	P1	P2
**Average**	22.83	64.76	98.73	64.84	98.53	113.42	61.33	91.67	107.00
**Stdev**	0.86	8.23	8.31	8.05	7.60	8.00	6.29	5.03	-
**Min**	22.58	58.63	93.59	58.72	93.18	108.80	45.00	79.00	107.00
**Max**	23.04	69.18	102.04	69.28	101.87	117.72	75.00	99.00	107.00

Statistical measures (model) of parr fork lengths (mm) in May (alevin emergence) and at the end of the growth season. Size data are broken down per life phases and age groups. The tables report the averages and standard deviations over 100 years, averaged over 10 independent runs of the model. The overall minimum and maximum values over the 100 years and 10 independent runs are also reported. The results are compared with those of the 2010 survey in the Os river (Rådgivende Biologer AS, 2012).

**Table 9:** Scenario 1 –Average smolt fork length.

Date	01-November			31-April			15-October		
Source	Model						Os river		
Age	Smolt0+	Smolt1+	Smolt2+	Smolt0+	Smolt1+	Smolt2+	Smolt0+	Smolt1+	Smolt2+
**Average**	92.20	109.65	128.35	115.42	129.11	147.55	-	112.33	128.26
**Stdev**	0.80	7.41	9.31	2.11	9.57	11.50	-	9.49	9.39
**Min**	0.00	104.23	122.29	0.00	117.79	137.46	-	100.00	110.00
**Max**	100.44	114.10	132.67	135.91	138.46	155.87	-	137.00	147.00

Statistical measures (model) of smolt fork lengths (mm) at the beginning and end of the smolt sub-phase. Size data are broken down per life phases and age groups. The tables report the averages and standard deviations over 100 years, averaged over 10 independent runs of the model. The overall minimum and maximum values over the 100 years and 10 independent runs are also reported. The results are compared with those of the 2010 survey in the Os river (Rådgivende Biologer AS, 2012).

**Table 10:** Scenario 1 –Average oceanic salmon fork lengths (mm) and weights (g).

Date	31-September							
Source	Model							
Measure	Length				Weight			
Age	0SW	1SW	2SW	3SW	0SW	1SW	2SW	3SW
**Average**	352.14	606.05	797.90	999.28	539.68	2731.46	6210.86	12164.21
**Stdev**	19.25	39.98	63.39	99.14	87.66	534.41	1458.50	3546.47
**Min**	307.83	531.94	706.52	880.94	361.14	1850.20	4318.83	8347.94
**Max**	390.64	691.51	901.51	1120.49	735.70	4050.58	8943.78	17123.50

Statistical measures of oceanic salmon fork lengths (mm) and weights (g). Size data are broken down per life phases and age groups. The tables report the averages and standard deviations over 100 years, averaged over 10 independent runs of the model. The overall minimum and maximum values over the 100 years and 10 independent runs are also reported.

**Table 11:** Scenario 1 –Average spawners fork lengths (mm).

Date	01-October			-		
Source	Model			N. Johnson et al. (1991)		
Age	1SW	2SW	3SW	1SW	2SW	3SW
**Average**	602.36	791.99	989.39	611.45	821.97	970.33
**Stdev**	21.89	24.83	33.08	63.87	74.94	77.23
**Min**	527.00	703.45	878.06	554.10	715.00	823.30
**Max**	688.40	895.78	1103.57	690.00	903.20	1028.00

Statistical measures of spawners fork lengths (mm). Size data are broken down per life phases and age groups. The tables report the averages and standard deviations over 100 years, averaged over 10 independent runs of the model. The overall minimum and maximum values over the 100 years and 10 independent runs are also reported. The results are compared with those of N. Johnson’s et al’s (1991) survey in seventeen Norwegian rivers.

For juvenile salmon, the results of the simulations are compared to the results of the 15-10-2010 survey in the river Os [39]. In the river, no young-of-the-year smolts were found. All except one p2 had smolted.

As detailed numbers for adult weights were lacking for the river Os, we took the results of the survey by Johnson et al. [40] on 17 Norwegian rivers, and calculated the average and spread of the data.

Overall, the statistics of the populations generated by IBSEM coincide with data from natural populations, including the range of values for salmon populations reviewed Hutchings and Jones [85]. These authors found that the average length of p1 in Norwegian rivers is 83±9 mm (65±8 mm in May and 92±5 mm in November in the model), average smolt length 131±12 mm (129±10 mm and 147±12 mm at migration for respectively 1+ and 2+ smolts in the model), and average grilse length 595±43 mm (602±22 mm in the model).

Given the average number and weight of spawners (Tables 3 and 11), the total weight of returning salmon can be estimated to about 2000 kg. In 2011, 353 salmon were captured by fishermen in the river Os, for a total weight of 1200 kg. The total number of returners to the river was estimated to 525 salmon (67% exploitation rate), giving a total weight close to the result of the simulations.

**Fig 3:**
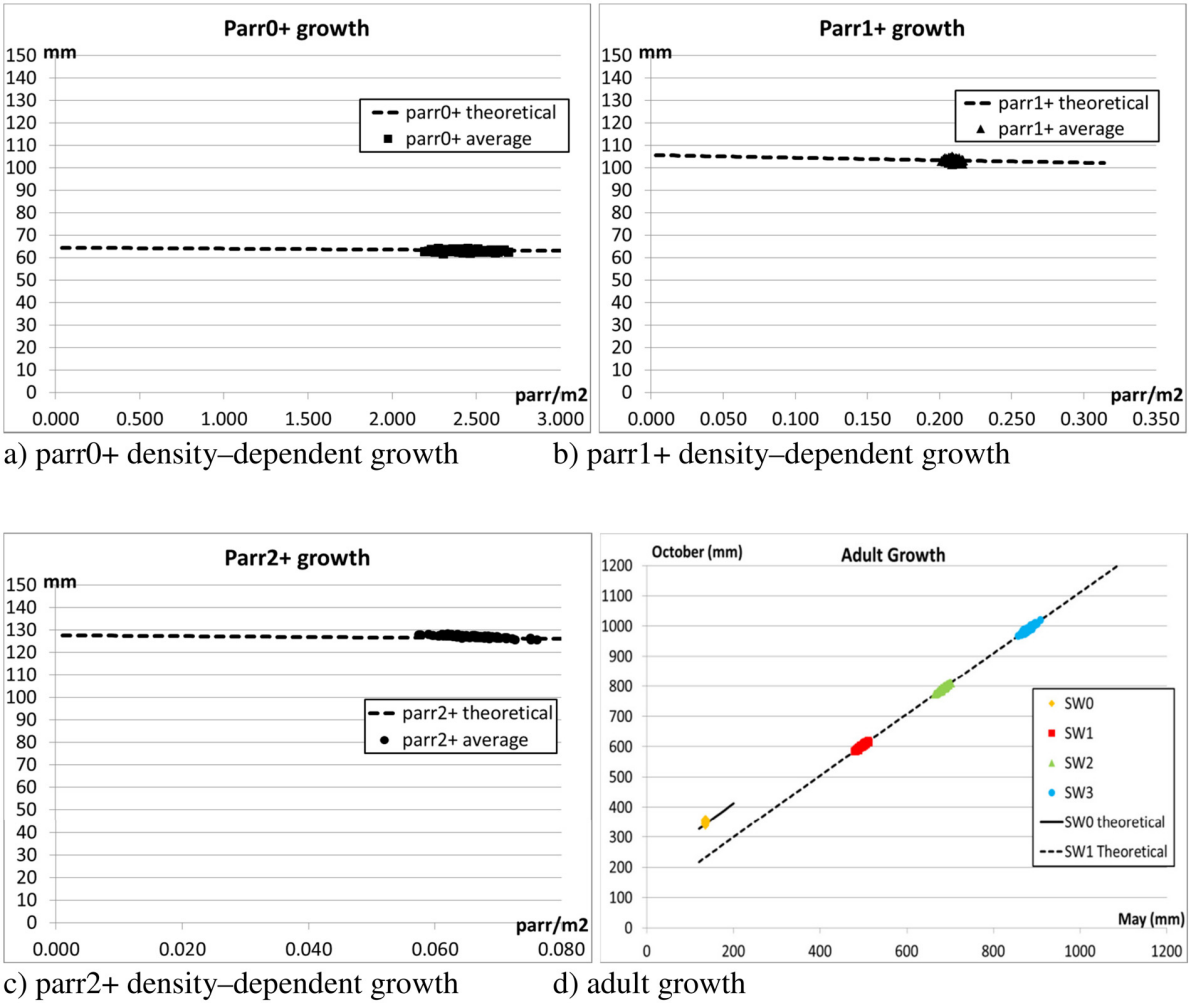
Scenario 1—Juvenile and adult growth. The theoretical curves for density-dependent juvenile growth (default settings as in Table B in S2 File) are plotted together with the results of the simulations. Figs a)-c) show the average fork length of parr at end of the growth season (31-October) against the density on 01-May (alevin emergence). The theoretical curves for adult growth (default settings as in Table B in S2 File) are plotted together with the results of the simulations. Fig d) shows the average size per year class on 31-September (before spawners return to freshwater) against the size on 01-May (migration of smolts to sea). The Figs show the average values of 10 independent runs, and plot the distribution of the results over the 100 monitoring years.

Fig 3 shows that the results of the simulations closely follow the theoretical curves for density-dependent fork length of juvenile salmon at the end of the growth season against the density in May. Fig 3 also shows a close correspondence between the actual and expected size of adult salmon at the end of September versus the initial size in May.

#### 3.1.4 Scenario 1 –emergent population, mortality

**Fig 4:**
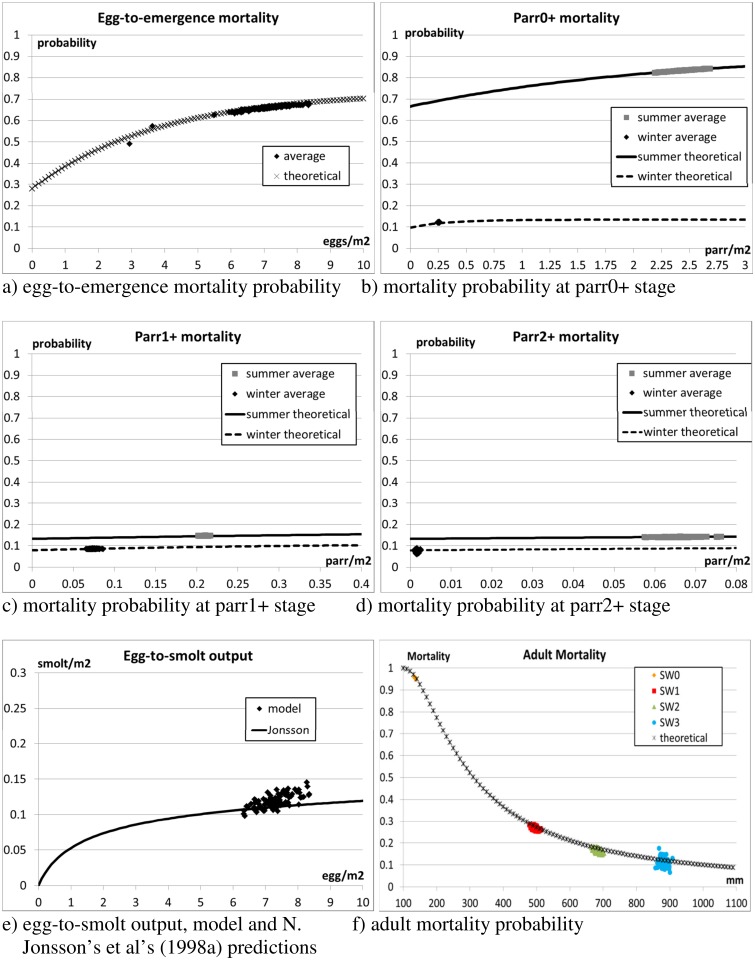
Scenario 1—Juvenile and adult mortality. The theoretical curves for density-dependent juvenile and size-dependent adult mortality (default settings as in Table C in S2 File) are plotted together with the results of the simulations. Fig e) compares the simulated egg-to-smolt output with the density-dependent curve theorised by N. Jonsson et al. (1998a). The Figs show the average values of 10 independent runs, and plot the distribution of the results over the 100 monitoring years.

Fig 4 shows the actual (simulations) and expected (theoretical curves from model equations) mortalities. The results of the simulations match very well the theoretical curves. The experimental results are detailed in Tables 11–14.

**Table 12:** Scenario 1 –Statistical measures of egg mortality.

Phase	Egg-to-alevin	Egg-to-smolt
Source	Model	Model
**Average**	0.664	0.984
**Stdev**	0.020	0.002
**Min**	0.592	0.973
**Max**	0.714	0.990

Mortality data are broken down per life phases and age groups. The tables report the averages and standard deviations over 100 years, averaged over 10 independent runs of the model. The overall minimum and maximum values over the 100 years and 10 independent runs are also reported.

**Table 13:** Scenario 1 –Statistical measures of juvenile mortality.

Date	01-May / 31-September			01-November / 28-February			01-November / 31 April
Source	Model			Model			Model
Age	P0	P1	P2	P0	P1	P2	Smolt
**Average**	0.833	0.147	0.141	0.122	0.085	0.075	0.168
**Stdev**	0.011	0.002	0.003	0.002	0.003	0.015	0.003
**Min**	0.801	0.141	0.130	0.116	0.077	0.008	0.159
**Max**	0.868	0.155	0.152	0.128	0.093	0.134	0.176

b) Parr mortality for period between 01-May (alevin emergence) and 31- September (before sneaker parr maturation), and for resting season. Smolt mortality for whole smolt sub-phase. Mortality data are broken down per life phases and age groups. The tables report the averages and standard deviations over 100 years, averaged over 10 independent runs of the model. The overall minimum and maximum values over the 100 years and 10 independent runs are also reported.

**Table 14:** Scenario 1 –Statistical measures of oceanic salmon mortality.

Date	01-May / 31-September				Returners	
Source	Model				Model	
Age	0SW	1SW	2SW	3SW	Smolt-to-grilse	Overall
**Average**	0.954	0.266	0.164	0.111	0.0078	0.0144
**Stdev**	0.002	0.007	0.008	0.018	0.0018	0.0023
**Min**	0.948	0.253	0.146	0.066	0.0034	0.0084
**Max**	0.958	0.296	0.192	0.180	0.0181	0.0244

Oceanic salmon mortality for period between 01-May (migration to sea) and 31-September (before maturation). Survivorship for the whole oceanic phase is estimated for grilse and overall (total returners per year class of smolts) population. Figures are broken down life phases and age groups. The tables report the averages and standard deviations over 100 years, averaged over 10 independent runs of the model. The overall minimum and maximum values over the 100 years and 10 independent runs are also reported.

Mortality in the river Os was estimated to 87% from egg to young-of-the-year (p0) in autumn [39]. Adding up egg-to-emergence mortality (Table 12) with p0 mortality from May to October (Table 13), we estimated 94.4% mortality in the simulations from egg to p0. Considering that the model stabilises on higher population densities than those estimated in the river, it can be concluded that the mechanisms determining density-dependent mortality rates in the river are simulated effectively. The adult return rate from oceanic migration was estimated to 1.1% for the population in the river Os in the period 1992–2008. 1.4% was obtained in the simulations here (Table 14).

Overall, the model gives mortality rates that are in good agreement with those reported in the river Os. The range of values of the overall egg-to-smolt density relationship (Fig 4e) is also reasonably close to the theoretical curve calculated by Jonsson et al. [83] for the salmon population of the river Imsa.

#### 3.1.5 Scenario 1 –emergent population, maturation and smoltification

There are no available data on the percentage of pre-smolts per juvenile year class in the river Os. However, the good match between real and simulated overall smolt densities (Section 3.1.2) and lengths per year class (Section 3.1.3) indicates that size-dependent smolting has been successfully modelled.

In the simulations, approximately 40% and 90% of respectively p1 and p2 male parr were sexually mature at the beginning of October. These percentages closely mirror those (43% p1, 93% p2) found in the river Os during the October 2010 survey [39]. This result, together with the close correspondence in size at different ages between simulated and biological populations, suggests that the maturation probability curve (Section 2.3.3; Section A.4.3 in S1 File; Section B.2.3 in S2 File) is realistic.

#### 3.1.6 Scenario 1 –emergent population, genetic make-up

**Fig 5:**
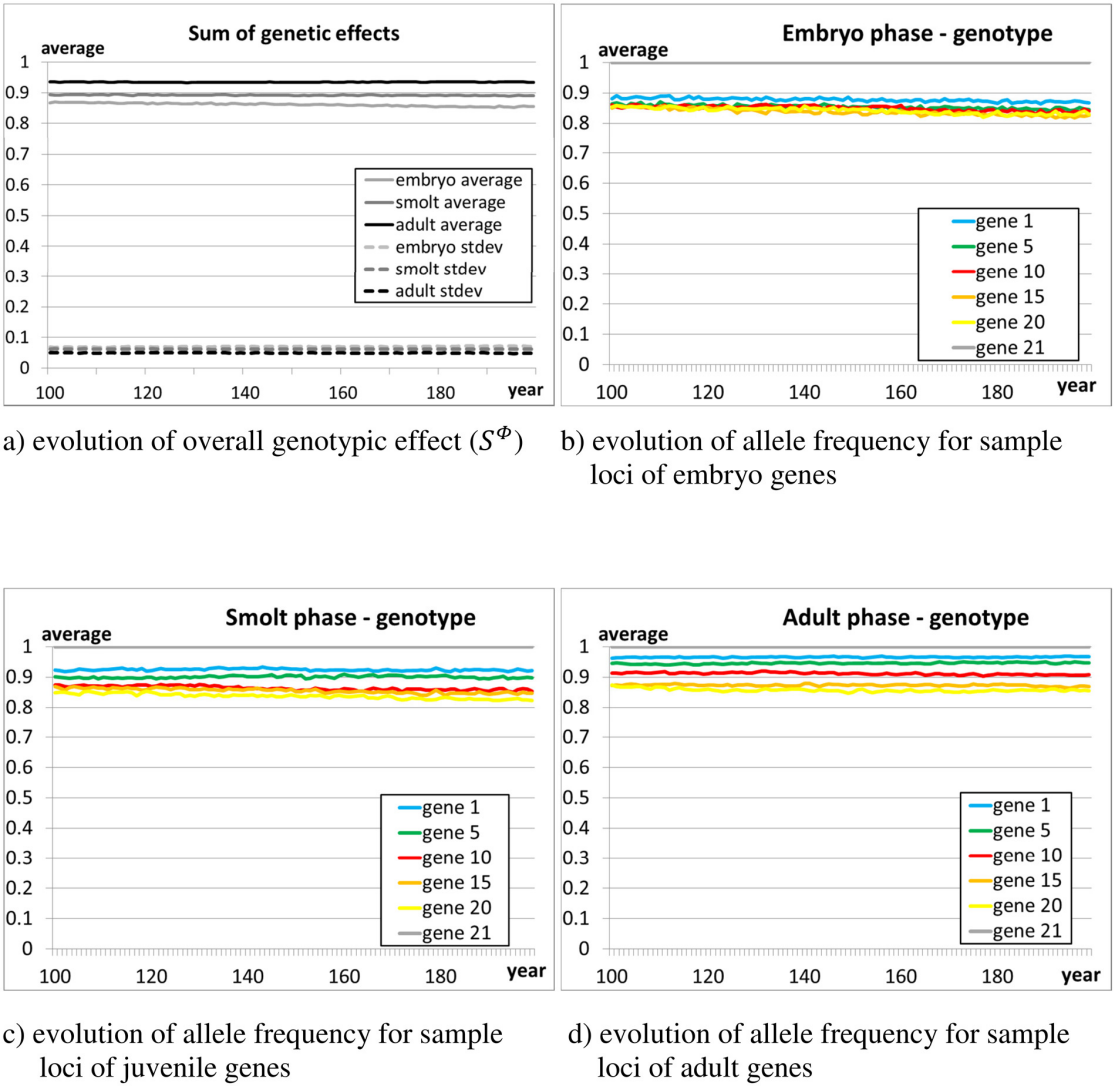
Scenario 1—Plots b)-d) show, for each set of genes (embryonic, juvenile, adult), the evolution of the alleles for a number of sample loci during the 100 monitoring years. The figures show the average allelic values of the population, averaged over 10 independent runs. A value of ‘1’ means that only the ‘wild’ allele is present, a value of ‘0’ means that only the farm allele is present. Plot a) shows the average of the overall genotypic effect (S^Φ^, Eq. A.2 in S1 File) on the traits of the three main phases (embryonic, juvenile, adult). The population was initialised as composed of farm individuals (average frequency of allele ‘1’ ≈0.1, S^Φ^≈0.1), and subjected to a simulated immigration of spawners of wild origin (strayers from other rivers, average frequency of allele ‘1’ ≈0.8, S^Φ^≈0.8, see Section 2.4.6) equal to 5% of the overall number of returners.

Fig 5b–5d shows the evolution of the allelic values for a sub-sample of six loci of the three sets of genes influencing the traits in the embryonic, juvenile, and adult phases. The immigration of slightly dissimilar strayers introduces additional genetic diversity in the population, and facilitates evolution away from the initial genetic composition in presence of a fitness gradient. The plots show the evolution over the 100 monitoring years, with a stable predominance of ‘wild’ alleles in the genetic make-up of the population.

#### 3.1.7 Scenario 1 –emergent population, stability

**Fig 6:**
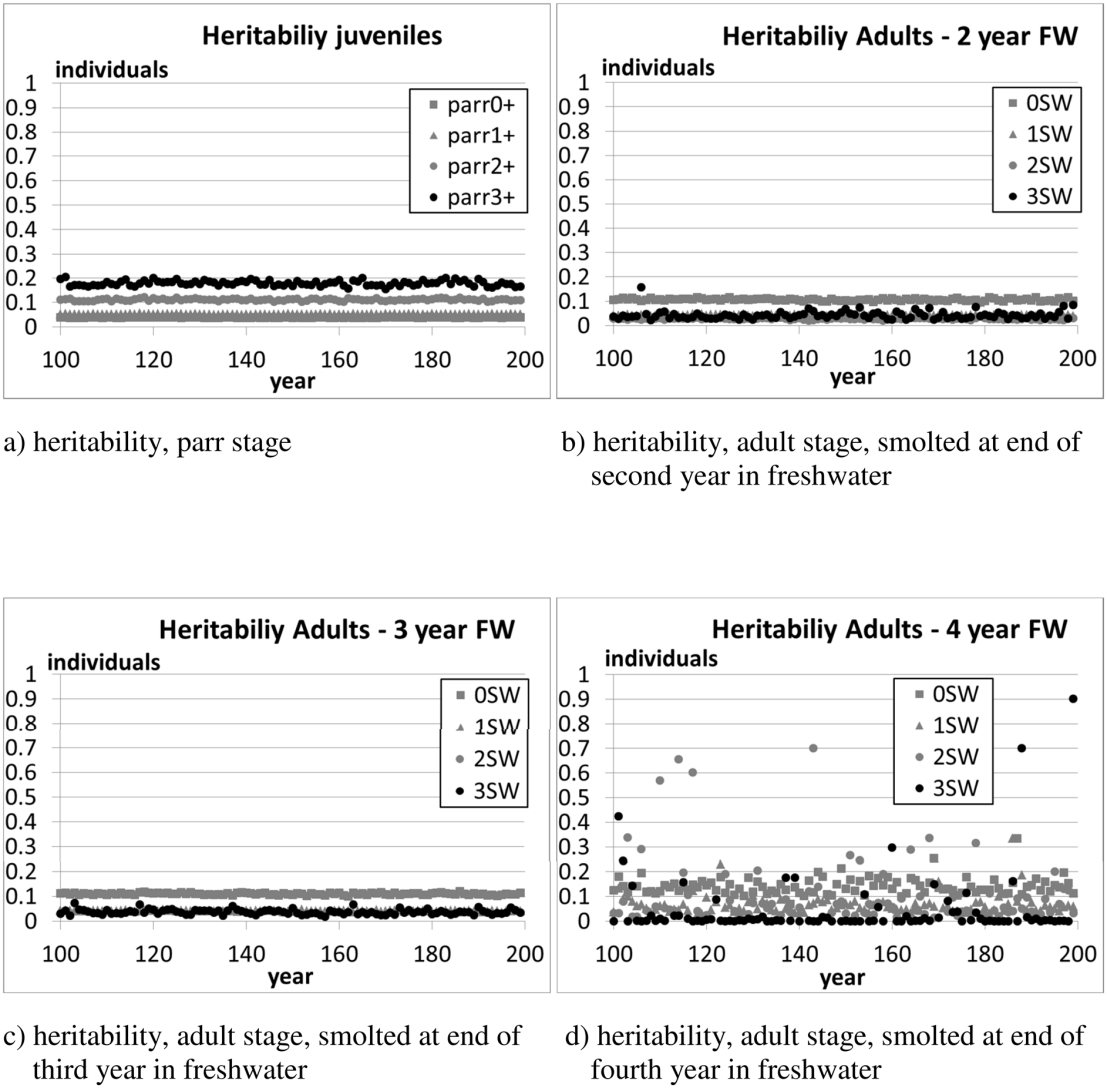
Scenario 1—The evolution of the heritability of the fork length (i.e. activity trait in Eq. 11) in the simulated population. The figures show the average values of 10 independent runs over the 100 monitoring years. The population was initialised as composed of farm individuals (average frequency of allele ‘1’ ≈0.1, S^Φ^≈0.1), and subjected to a simulated immigration of spawners of wild origin (strayers from other rivers, average frequency of allele ‘1’ ≈0.8, S^Φ^≈0.8, see Section 2.4.6) equal to 5% of the overall number of returners.

Fig 5a shows the evolution of the population average and standard deviation of the sum of genetic effects on traits (S^Φ^, Eq. A.2 in S1 File) for the three gene sets (embryonic, juvenile, adult) characterising the three main life phases. For each set of genes, 0≤S^Φ^≤1, with S^Φ^≈0.9 in individuals of wild origin and S^Φ^≈0.1 in individuals of farm origin. The trajectories are flat, indicating a stationary distribution. The non-negligible spread (standard deviation) of S^Φ^ across the population proves the persistence of genetic diversity in the population. The genetic diversity of the population is also manifested as non-negligible heritability (h^2^) of the fork length (Fig 6). Overall, Figs 5 and 6 demonstrate that the wild salmon population is in an evolutionary equilibrium.

### 3.2 Scenario 2 –Test of the Genetic Model

If IBSEM is to be used to study the effects of changes in the genetic profile of a salmon population, it is important to verify that the evolutionary equilibrium of the system corresponds with a population of wild salmon. That is, we need to verify whether the genetic configuration of mainly wild (‘1’) alleles corresponds to a global evolutionary attractor in absence of introgression. This is to guarantee that the response to introgression of different genotypes, for example, escaped farmed salmon, is not confounded with the response to natural selection. Therefore, in scenario 2, the population is initialised with a radically different genetic make-up, and its genetic composition is monitored to verify whether it evolves towards genotypes composed of mainly wild alleles.

#### 3.2.1 Scenario 2 –model initialization

IBSEM is parameterized as in scenario 1 (Section 3.2.1), with the only exception that the population is randomly initialised with a ‘farm’ genotype (0.1 frequency of wild ‘1’ alleles). The evolution of the genetic make-up of the individuals is monitored over the whole 200 years of evolution, and the results are averaged over 10 replicates. The genetic composition of the embryo population is monitored at the end of April, before alevin become parr (Section 2). The genetic composition of the juvenile population is sampled from the pool of pre-smolts, when they begin to differentiate in size from other parr at the beginning of November (Section 2.3.4). The genetic composition of the adult population is monitored at the end of September, before mature individuals return to spawn (Section 2.3.3).

#### 3.2.2 Scenario 2 –genotype of emergent population

**Fig 7:**
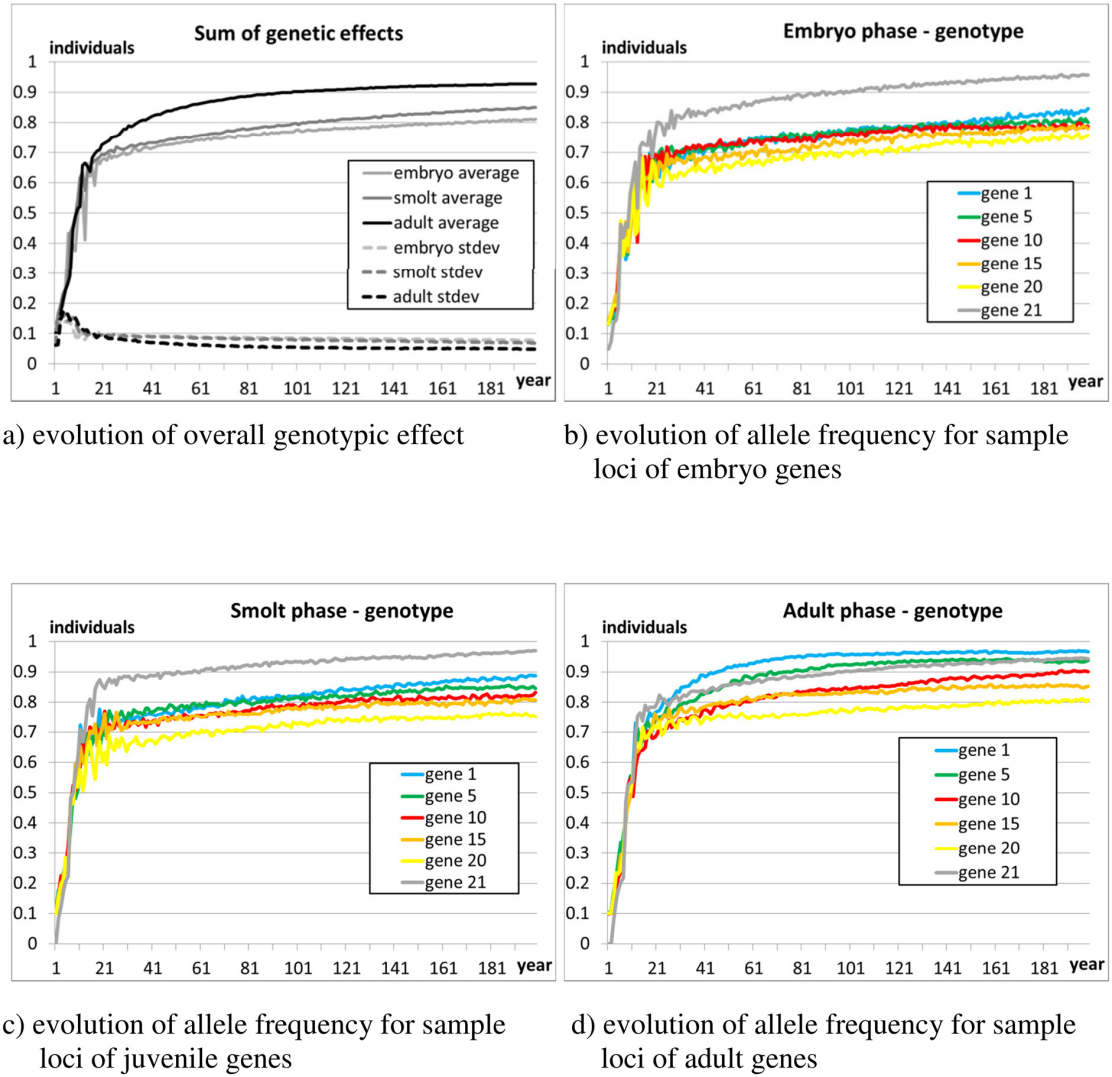
Scenario 2—Plots b)-d) show, for each set of genes (embryonic, juvenile, adult), the evolution of the alleles for a number of sample loci during the 200 evolution years. The figures show the average allelic values of the population, averaged over 10 independent runs. A value of ‘1’ means that only the ‘wild’ allele is present, a value of ‘0’ means that only the farm allele is present. Plot a) shows the overall genotypic effect (S^Φ^, Eq. A.2 in S1 File) on the traits of the three main phases (embryonic, juvenile, adult). The population was initialised as composed of farm individuals (average frequency of allele ‘1’ ≈0.1, S^Φ^≈0.1), and subjected to a simulated immigration of spawners of wild origin (strayers from other rivers, average frequency of allele ‘1’ ≈0.8, S^Φ^≈0.8, see Section 2.4.6) equal to 5% of the overall number of returners.

Fig 7 is analogous to Fig 5 and shows that the population converges to substantially ‘wild’ genotypes in approximately 150 years. The average genotypic value S^Φ^ settles beyond the 0.8 value of the strayer genotype, and approximates the default 0.9 wild value.

The rise in S^Φ^ is faster in the adult phase, where action of natural selection is most prolonged (at least one year at sea). Due to their size, fast growing young-of-the-year males of farm origin have good chances to mature (Section 2.3.3). Due to this ‘shortcut’ to reproduction, low-fitness farm alleles are able to persist longer in the population, and the average of the sum of genetic effects rises more slowly in juveniles than in adults. This result partly agrees with Hindar’s et al.’s [86] postulation that sexual maturation in parr increases the success of individuals of farm origin. Change in the sum of genetic effects is slowest in the embryonic gene set. This is due to the short duration of the embryonic phase, which limits the effect of natural selection.

Locus 21 is the neutral marker (section 2), and is equal to ‘0’ in individuals of farmed origin and ‘1’ in wild salmon (including strayers). Its evolution (Fig 7) shows the speed at which wild genotypes (strayers) invade and replace the initial farmed population.

**Fig 8:**
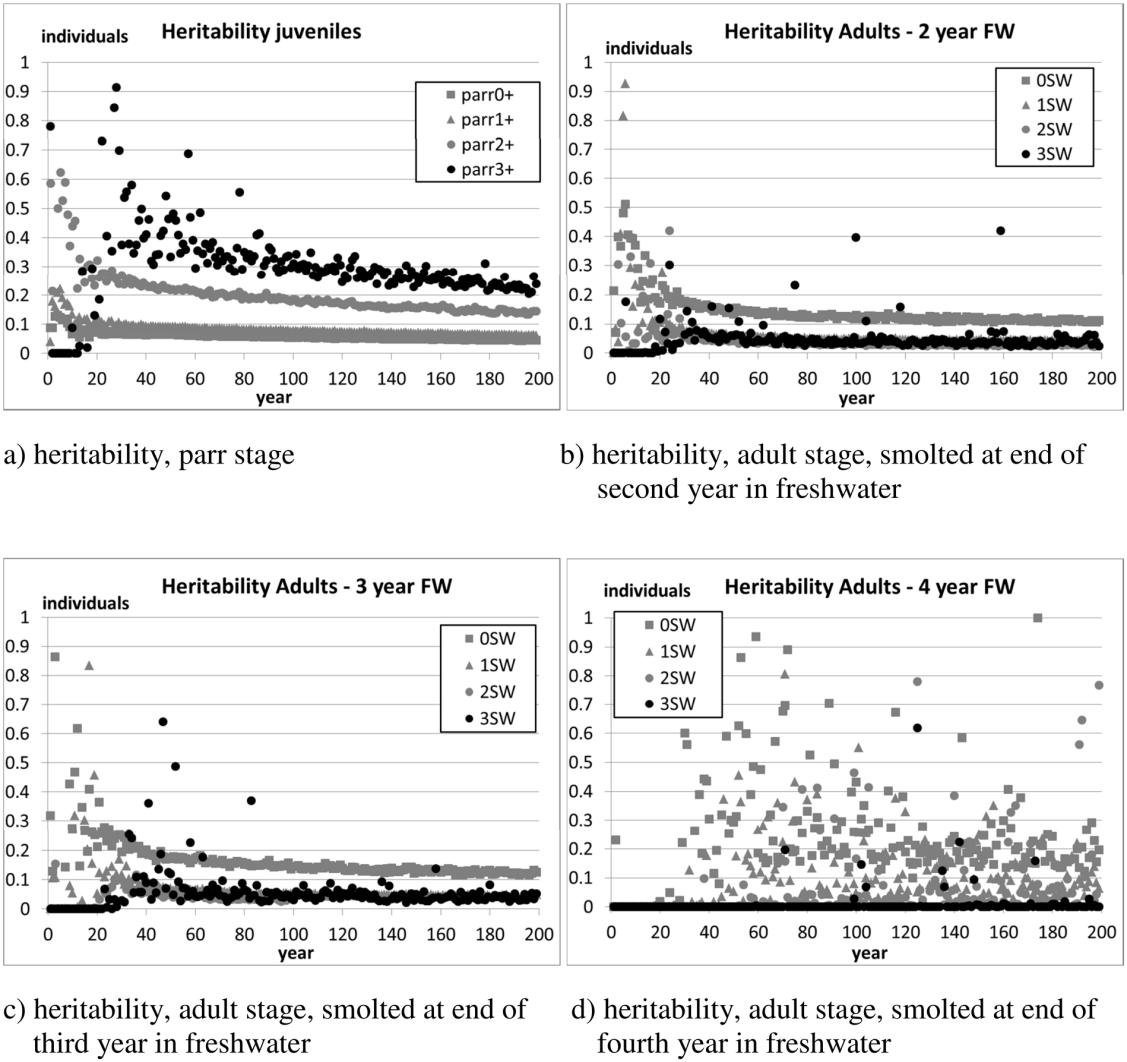
Scenario 2—The evolution of the heritability of the fork length in the simulated population. The figures show the average values of 10 independent runs over the 200 evolution years. The population was initialised as composed of farm individuals (average frequency of allele ‘1’ ≈0.1), and subjected to a simulated immigration of spawners of wild origin (strayers from other rivers, Section 2.4.6) equal to 5% of the overall number of returners.

Fig 8 is akin to Fig 6, and shows that, after 200 years of evolution, the heritability of the fork length converges to a value similar to that of a wild population.

### 3.3 Scenarios 3, 4, 5 –Tests of Sensitivity

In parameterizing the model, several assumptions, based upon values from empirical studies, were made concerning the differences between fish of farm and wild origin. The two most significant ones regard higher growth of farmed fish ([20][21][22][23][27]) and their mortality in the wild ([25][26][7][27]). These assumptions affect the introgression rate of farmed salmon into wild populations. Scenarios 3, 4 and 5 test the sensitivity of the model to the said assumptions.

#### 3.3.1 Scenarios 3, 4 and 5 –Model initialization

IBSEM is parameterized as in scenario 1 (Section 3.2.1), with the only exception that the difference between the genotypic effect of wild and farm genes (i.e., the relative fitness difference) on growth and mortality is changed. In scenario 3 the standard setting (scenario 1) is used. In scenario 4 the genetic difference between farmed and wild salmon is halved (i.e., farmed and wild salmon have more similar growth and mortality rates), in scenario 5 the genetic difference is doubled (i.e., farmed and wild salmon differ more in growth and mortality). In all scenarios, the wild population is first let to settle for 50 years without intrusion of farm fish (settling phase). After that, farm individuals are introduced into the local population for 200 years (introgression phase). Finally, introgression is stopped and the population is let to recover for another 200 years (recovery phase).

In the previous tests, 5% of returners were assumed to stray into neighbouring rivers and were replaced by randomly initialised wild individuals (0.8 frequency of wild allele). Preliminary tests showed that the straying of wild individuals has a buffering effect on the results of the introgression of farmed individuals. That is, the introduction of strayers of mainly ‘wild’ genotype partly counterbalances the introduction of fish of mainly ‘farm’ genotype. To highlight the consequence on fitness of the assumptions about the influence of genes of wild and farm origin, the genotype of the incoming strayers is made similar to that of the local population. That is, each returner straying into another river is replaced by a strayer from another river. The genotype of the incoming strayer is randomly initialised with the same frequency of ‘wild’ alleles as the genotype of the outgoing strayer. In this way, straying still supports the diversity without altering the allele frequencies in the population.

Three introgression cases are considered per scenario: mild, intermediate, and high. In the first case, 25 randomly initialised adult salmon of farm origin (0.1 frequency of wild allele) are added to the pool of spawners, in the second case 100 spawners of farm origin are added, and in the third case 250 spawners of farm origin are added. The three introgression rates roughly correspond to respectively 5%, 20%, and 50% of the average pool of spawners obtained in the simulations of scenario 1 (i.e., the stabilised wild population prior to any introgression of farmed escapees). Ten independent runs were performed per each case, and the results were averaged.

#### 3.3.2 Scenarios 3, 4 and 5 –Effects of introgression

**Fig 9:**
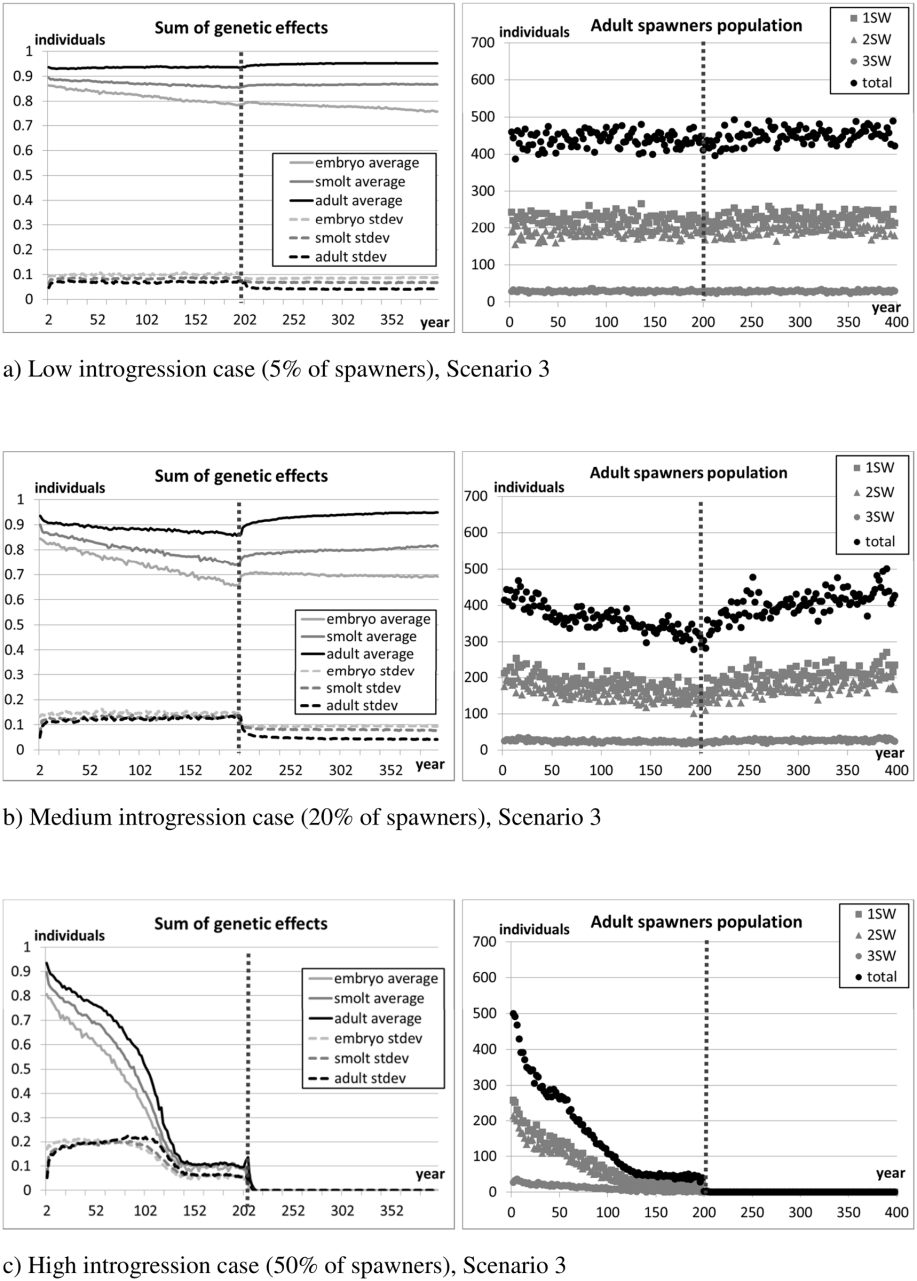
Scenario 3 –Standard parameter setting. Sum of genetic effects and size of spawners population. The vertical dotted line marks the stop of the introgression phase and the beginning of the recovery phase. The initial settling phase is not shown in the figure.

**Fig 10:**
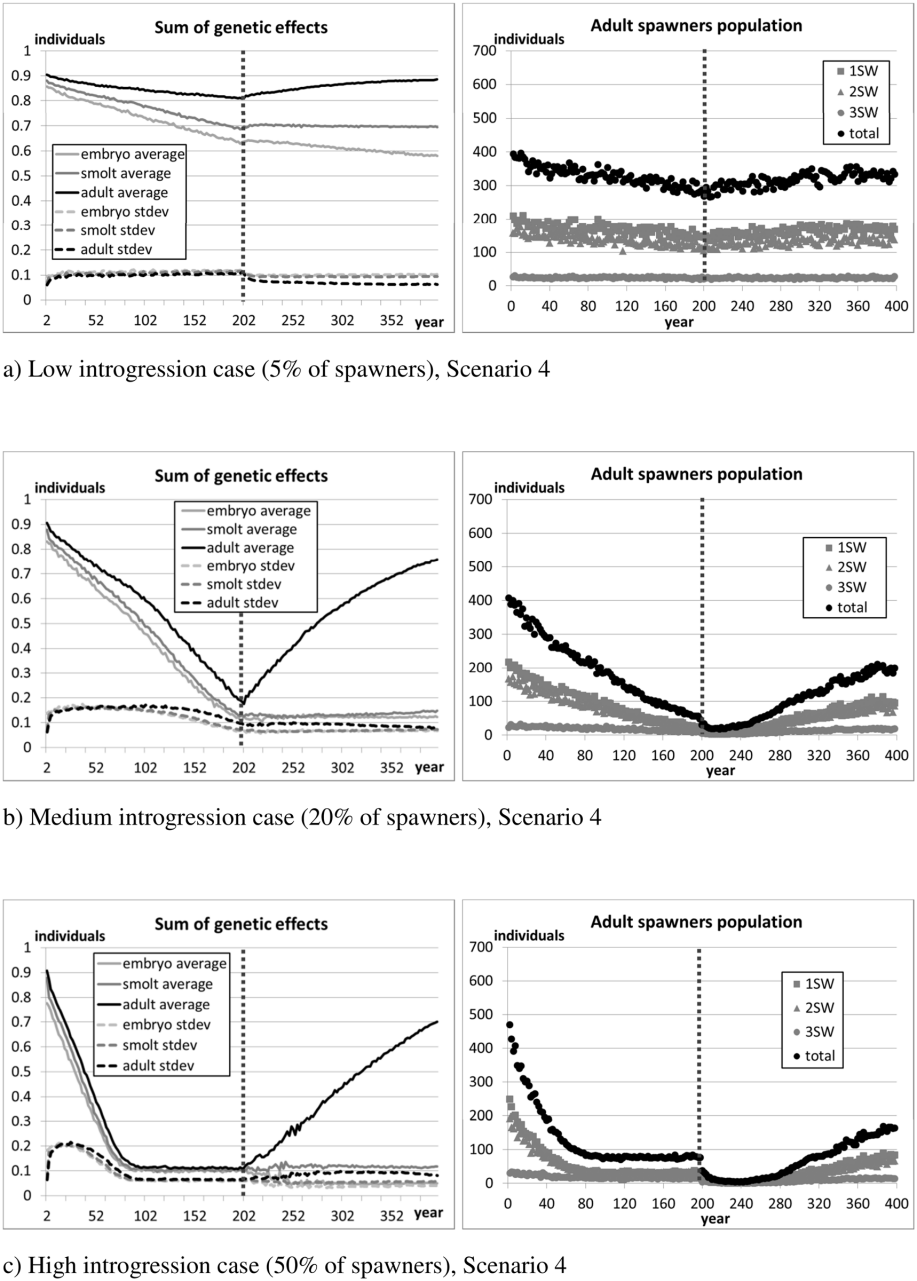
Scenario 4 –Differences in growth and mortality between salmon of farm and wild origin are halved. Sum of genetic effects and size of spawners population. The vertical dotted line marks the stop of the introgression phase and the beginning of the recovery phase. The initial settling phase is not shown in the figure.

**Fig 11:**
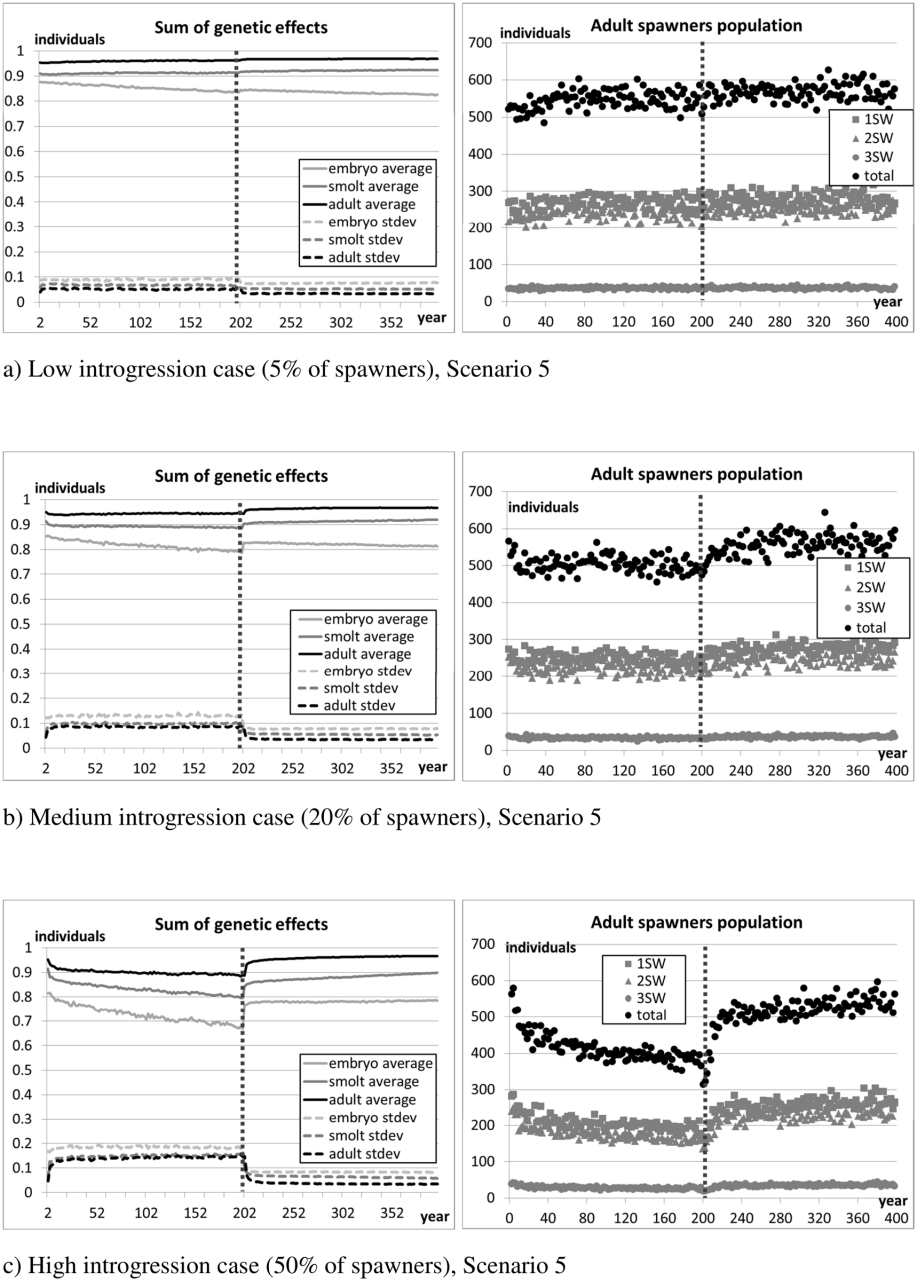
Scenario 5 –Differences in growth and mortality between salmon of farm and wild origin are doubled. Sum of genetic effects and size of spawners population. The vertical dotted line marks the stop of the introgression phase and the beginning of the recovery phase. The initial settling phase is not shown in the figure.

**Fig 12:**
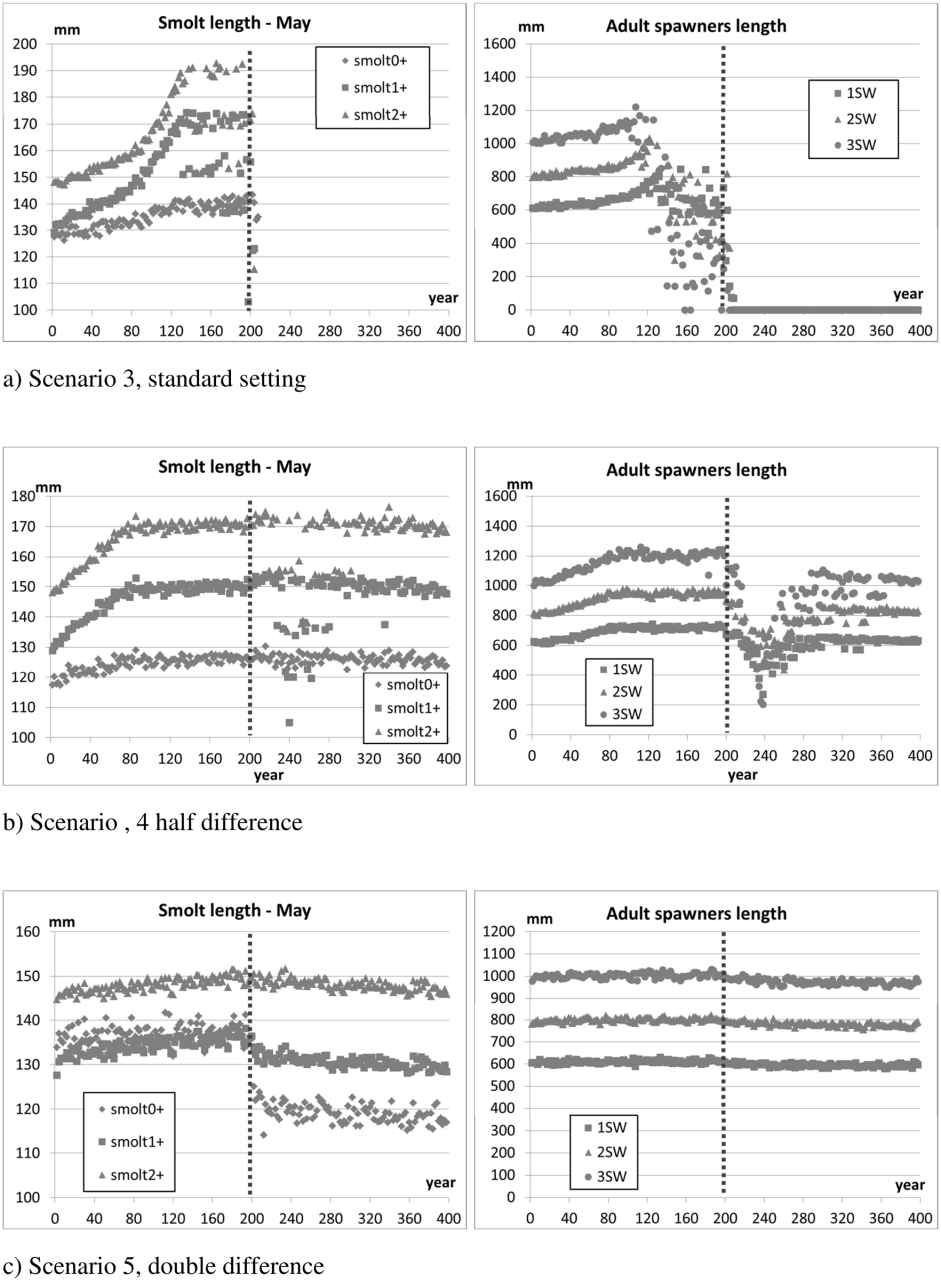
Scenario 3, 4, 5 –High introgression case (50% of spawners). Size of individuals: smolts are measured in May at the time of their migration to sea, adult spawners are measured before reproduction. Three tests with different rates in growth and mortality between salmon of farm and wild origin. The vertical dotted line marks the stop of the introgression phase and the beginning of the recovery phase. The initial settling phase is not shown in the figure.

Fig 9 (standard parameter setting), Fig 10 (halved differences), and Fig 11 (double differences) show the evolution of the average and standard deviation of the sum of genetic effects on traits (S^Φ^, Eq. A.2 in S1 File) and the average number of returners per year class over the 10 replicates. Fig 12 reports the average lengths of smolts (measured in May before migration) and adult spawners (before reproduction). The figures show the 200 years of introgression followed by the 200 years of recovery, ignoring the initial 50 years settling period.

As expected, introgression of farmed salmon is greatest when the genetic differences between farm and wild individuals are smallest (scenario 4, Fig 10). Conversely, introgression is lowest when the genetic difference between wild and farm individuals is largest (scenario 5, Fig 11). In all scenarios examined, introgression of farm genes is always associated with a drop in the number of returners in the focal wild population.

In the high introgression case of scenario 3 (standard setting), once introduction of farm individuals is stopped the remaining population is not viable and collapses (Fig 9). As the gene pool is progressively contaminated by farm genes, individuals increase their growth rate, and the average length of the population increases (Fig 12b). It is important to point out, however, that the increase in size of smolts and spawners is modest in the first 50 years following introgression. This result may point out the difficulty of timely detection of potentially catastrophic changes in the genetic make-up of a population by examining phenotypic traits alone.

In scenario 4 (fitness differences halved), due to the limited genetic difference between fish of farm and wild origin, the ability for natural selection to purge the farmed genes out of the population is weak. For this reason, farm genes spread rapidly during the 200 years introgression phase, and remain in the population thereafter. However, the resultant population, which is heavily contaminated by farmed escapees, is sufficiently fit to maintain its viability even in the high introgression case and after introgression is halted (scenario 4, Fig 10e). In contrast, in scenario 5 (fitness differences doubled), hybridisation is minimal in all introgression cases (i.e., low medium and high), and natural selection quickly purges farm genes from the population once the introduction of farmed individuals is stopped.

Where the differential between farmed and wild salmon is modest or weak (scenarios 3 and 4), the number of returners appears to be mostly correlated with the composition of the set of genes determining the traits in the adult phase. On the one hand, from the time they migrate to sea (May) to when they come back to spawn (October, one or more years later), returners need to survive at least 18 months at sea. Also, individuals of predominantly farm genotype are more likely to mature later than wild salmon (Section 2.4.3), and thus need to survive longer in the oceanic phase. On the other hand, fast growing salmon of mainly farm genotype are more likely to reach a size suitable for smolting earlier than wild salmon, and thus move faster to the next life phase, reducing exposure to freshwater mortality. Due to their size, they are also more likely to mature (and transmit their genes) as a precocious parr. The above reasons might explain the stronger selection and effect of wild alleles in the adult set of genes.

## 4. Discussion

IBSEM is a detailed eco-genetic model for Atlantic salmon. It was parameterized to simulate the life cycle of a wild salmon population in the river Os, Norway. Sensitivity analyses demonstrated that the abundance and structure of the salmon population produced by IBSEM closely matched empirical values observed in the river Os. Furthermore, the modelled values overlapped and were within the range of empirical values observed for a range of other Atlantic salmon populations. The model gave highly consistent results, and the emergent salmon population achieved an evolutionary equilibrium with a genetic profile that is close to our definition of the ‘wild’ genotype. We also showed that our definition of a wild genotype represents a global evolutionary attractor for the population. We conclude that the model is suitable for addressing a wide range of challenges to the demographic and evolutionary status of Atlantic salmon populations.

Due to the detail of its genetic and biological representation, and the consistency of the predictions, IBSEM represents a good tool to investigate different anthropogenic disturbances to simulated salmon populations, such as the documented intrusion of escaped farmed salmon [10][11][12]. As showed in our tests, the results of the simulations are sensitive to the range of documented genetic differences reported between wild and farm salmon ([7][20][21][22][23][25][26][27]), and give intuitively sensible results. For example, when the farmed salmon were set to be genetically more dissimilar and therefore display a greater fitness difference to the wild salmon, introgession rates were lower than when they were more genetically similar. However, the resultant changes in the demographic characteristics of the focal population were higher when the genetic difference between farmed and wild salmon was high. Similarly, differences between fish of different origin can be reduced or magnified by changing the frequencies of ‘1’ and ‘0’ alleles in farm and wild individuals. Such critical parameters can be adjusted to investigate and model the evolutionary fate of different populations and intrusion scenarios. This is important as empirical data from release experiments has demonstrated that the relative difference in fitness and survival of farmed, hybrid and wild salmon varies between rivers and environments [7][26][27].

The simulations also showed that straying from a neighbouring wild population has a buffering effect on the level of introgression of farm genes in the focal wild population. In scenarios 3–5 we made strayers genetically similar to the focal population. Thus, as the focal population became introgressed, the wild strayers were similarly introgressed too. This assumes that rivers in a given area are potentially affected by similar intrusion of farm individuals. While empirical evidence suggests that introgression of farmed escapees is strongly population dependent [11][12][86], it is clear that if the strayers were held as 100% wild, the buffering effect observed on the focal wild population would have been even greater.

One of the first Atlantic salmon full-life cycle models was created by Hindar et al. [86]. That model is based on a population of fixed size, and a number of vectors defining the survival and spawning success of individuals at different stages based on origin. The model does not use any explicit representation of the genotypes, and does not account for biological (e.g. individual size, population density) and environmental (e.g. temperature) factors on survival probabilities. Therefore, while the model by Hindar et al. [86] has been used to predict levels of introgression in native populations facing gene-flow from farmed escapees, it cannot be used to investigate potential biological and demographic changes in these populations. The need to predict potential functional changes in populations where farmed salmon have introgressed is urgent and vitally important [87][88].

Piou and Prevost [36] introduced IBASAM, a detailed eco-genetic model for salmon populations. IBASAM bears several similarities with IBSEM, particularly in the representation of the salmon life history. Compared to IBSEM, IBASAM includes the river flow in the representation of the environmental conditions, but does not take into account density-dependent survival. The main difference between the two models, however, is in the detail and extent of the genetic representation. In Piou’s and Prevost’s model only the maturation threshold is inherited. In our IBSEM model, the genetic make-up affects the entire life history (growth, mortality, and maturation) of the individuals at all life phases (embryonic, juvenile, adult). Moreover, IBSEM allows modulating the distribution of genetic effects on traits across the number of loci. This feature allows a richer and more realistic representation of salmon genetics than in IBASAM. Finally, the heritability of traits in the proposed model is an emergent property of the simulated system, and not forced externally via a fixed parameter like in IBASAM. Gilbey and Verspoor [34] and more recently Hedger et al. [35] built individual-based population models of salmon. Their models included life-history processes as well as environmental variables. However, both models are limited to the ecological representation of the population and do not simulate genetic effects on individual traits.

IBSEM was parameterized to fit experimental data and widely used theoretical curves. Given the interactions among the various variables (e.g. size influences mortality and maturation and smolting in parr), and their cascaded effects (e.g. changes in egg mortality affect the density and hence mortality and growth in parr), parameter fitting took a long time and several iterative adjustments were required. Nevertheless, IBSEM outputs reproduced the complexity of the salmon population in the river Os [39], and the range of values observed in other salmon rivers [40]. It is therefore suggested that the parameterization of IBSEM is both realistic and relevant for modelling evolutionary processes in Atlantic salmon populations faced with various challenges. Nevertheless, there are biological factors that have not yet been addressed in the model (e.g., the phenology of life-history transitions). Modelling such variables together with further environmental and anthropogenic parameters such as river flow, diseases, and angling pressure fisheries could add further accuracy and predictive power to the system. However, such potential adjustments will have to be traded-off with the increased complexity of the model. Nevertheless, the proposed individual-based system allows testing the consequences of new biological assumptions.

Despite the fact that evidence of likely negative effects of introgression of escaped farmed salmon in native populations is gradually building, thus far, there are no published studies demonstrating clear biological changes in native salmon populations as a direct result of the introgression of farmed salmon. Although this clearly does not mean that biological changes have not occurred, it is likely that the complexity of demonstrating such changes in native populations that may display large year to year fluctuations along with environmental conditions, and are exposed to a wide variety of potentially confounding challenges, makes unequivocally demonstrating such changes highly complex. This is where models, in particular IBSEM, can play a major role in predicting likely future outcomes.

## 5. Conclusions

In this paper we presented the individual-based eco-genetic salmon model IBSEM, and described its environmental, genetic, and biological components. We parameterized the model to simulate the life cycle of a wild salmon population based in the river Os in Norway, and migrating to the Norwegian sea during its oceanic phase. The range of biological outputs from the model were well within the range of values observed in the river Os as well as in a variety of other Atlantic salmon populations in Norway [40], and thus, the model is deemed representative of a range of Norwegian salmon populations. We proved the consistency of the model output, and documented its sensitivity to our assumptions on the fitness differences between individuals of farm and wild origin. We also showed that the emergent salmon population is in an evolutionary stable equilibrium with a genetic profile that is close to our definition of ‘wild’ genotype. IBSEM will permit simulating farmed salmon intrusions in order to identify the type and magnitude of biological changes one may expect to see under a large range of scenarios.

## Supporting Information

S1 File The Model. (DOCX)

S2 File Parameterization of Model. (DOCX)

S1 Minimal dataset (ZIP)
