# Supplementary material for: IBSEM: An Individual-Based Atlantic Salmon Population Model
Source: PLoS One. 2015 Sep 18;10(9):e0138444. doi: 10.1371/journal.pone.0138444 (PMC4575158; doi:10.1371/journal.pone.0138444)
Supplement: S1 File — (DOCX) [file pone.0138444.s001.docx]

**IBSEM: AN INDIVIDUAL-BASED ATLANTIC SALMON POPULATION MODEL**

Marco Castellani^1,2^, Mikko Heino^1,3,4^, John Gilbey^5^, Hitoshi Araki^6^, Terje Svåsand^1^, Kevin A. Glover^1^

^1^ *Institute of Marine Research, P.O. Box 1870, Nordnes, N-5817 Bergen, Norway*

^2^ School of Mechanical Engineering, University of Birmingham, Birmingham B15 2TT, UK.

^3^ *Department of Biology, University of Bergen, Bergen, Norway*

^4^ *IIASA, Laxenburg, Austria*

^5^ *Marine Scotland Science, Freshwater Laboratory, Faskally, Pitlochry, Scotland, U.K. PH16 5LB*

^6^ *Research Faculty of Agriculture, Hokkaido University, 060-8589, Japan*

# ONLINE S1 FILE

# THE MODEL

A.1 MODEL OVERVIEW

The Individual-Based Salmon Eco-genetic Model (IBSEM) reproduces the life cycle of an Atlantic salmon (*Salmo salar* L.) population. The model divides the life history of the individuals into three main phases: embryonic (egg to the end of endogenous feeding on its embryonic yolk-sac reserves, *E*), freshwater (juvenile, *J*), and oceanic (adult, *A*). Within each phase, the effect of environmental, genetic, and demographic factors is taken into account.

The embryonic phase covers the first month after emergence and includes two sub-phases: egg (*egg*) and alevin (*al*). The freshwater phase may last several years, and includes two main sub-phases: parr and pre-smolt (henceforth just named smolt, *sm*). The parr sub-phase is further divided into three age-related groups: young-of-the-year (*p0*), one year old (*p1*), and older than one year (*p2*). Similarly, the adult phase is divided according to the number of full winters spent at sea: first year at sea (*0SW*), one year (*1SW*), two year (*2SW*), and three or more years (*3SW*) at sea.

A year at the parr sub-phase is divided in a *growth season* (warm months) and a *resting season* (cold months) where growth halts and mortality decreases. Smolts do grow also during the resting season, albeit at a lower rate. In the oceanic phase there are no growth and resting seasons.

In the model, eggs hatch on the 1^st^ of April. On the 1^st^ of May, the surviving alevin are considered parr. Young-of-the-year parr grow until the 31^st^ of October, when they enter the resting season. Male parr may mature on the 1^st^ of October. Sexually mature parr do not grow in size during the month of October, in order to account for the costs of gonad development.

A.2 PHYSICAL ENVIRONMENT

Two variables are used to define the physical environment: the river area *S* and the average monthly temperature t_m_ of the water.

The temperature t_m_ at month $m\in\left\{ 1,2,\ldots,12 \right\}$ can be fixed to the pre-set value T_m_, or randomised to model temporal fluctuations. In the latter (default) case, the monthly temperature is sampled from a Gaussian distribution centred on T_m_ and having standard deviation$\sigma_{T}$:

t_m_ = gaussian(T_m_, $\sigma_{T}$) (A.1)

A.3 INHERITANCE

Individuals are diploid (i.e., with two sets of chromosomes), and their phenotype is defined by three independent sets of *L* genes. Each set of genes defines the traits of the individual in one of the three main life phases: embryonic, freshwater, and adult. Reproduction is sexual. The sex of the individual is randomly determined at birth, with an equal sex ratio. Individuals inherit a maternal and a paternal allele per locus at random.

For each trait, the genotypic effect is determined using a simple additive model, using an exponentially declining distribution of weights across the set of loci. That is, there are a small number of genes which have relatively large effects on the trait and an increasing number of other genes each having an exponentially smaller influence.

We use binary representation where each locus *l* ($l\in\left\{ 1,2,\ldots,L \right\}$) can take up one of the two alleles, ‘0’ and ‘1’, giving rise to three possible biallelic genotypes per locus (‘00’, ‘01’, and ‘11’).

Specifically, an individual’s total allelic value $S^{\Phi}$ is a real number between 0 and 1 defined for each life phase $\Phi\in\left\{ E, J, A \right\}$ as:

$S^{\Phi}=\frac{1}{L}\cdot\sum_{l=1}^{L} \epsilon_{l}\cdot\frac{g\left( \Phi,1,l \right)+g\left( \Phi,2,l \right)}{2}$ (A.2)

where $g(\Phi,j,l)$ is the binary allele value for locus *l* of the *j*^th^ copy of gene set $\Phi\in\left\{ E, J, A \right\}$. Genetic and phenotypic differences among farm and wild salmon are encoded through different allele frequencies. Wild salmon are initiated with frequency 0.9 of wild ‘1’ alleles ($g\left( \Phi,j,l \right)=1$), whereas individuals of farm origin are initiated with frequency 0.9 of ‘0’ alleles ($g\left( \Phi,j,l \right)=0$).

We assume that the magnitude $\epsilon_{l}$ of the genotypic effect of a given locus *l* on the traits follows the exponential distribution described in Fig. A:

$\epsilon_{l}=\frac{e^{-\Gamma\cdot l}}{\sum_{i=1}^{L} e^{-\Gamma\cdot i}}$ (A.3)

where $\Gamma$ is a system parameter. The last locus in a set of chromosomes has no phenotypic effect ($\epsilon_{L}=0$); this locus serves as a neutral marker and can be used to track the introgression in the population. In wild salmon the last locus is always initiated to the allele$g\left( \Phi,j,L \right)=1$, and in farmed salmon to$g\left( \Phi,j,L \right)=0$.

The allelic value $S^{\Phi}$ is mapped to the genetic value$\delta^{T}\left( \Phi\right)$ (also known as the breeding value) through Eq. (A.4):

$\delta^{T}\left( \Phi\right)=\delta_{w}^{T}\left( \Phi\right)+\left[ \delta_{f}^{T}\left( \Phi\right)-\delta_{w}^{T}\left( \Phi\right) \right]\cdot\frac{0.9-S^{\Phi}}{0.8}$ (A.4)

where $\delta_{w}^{T}\left( \Phi\right)$ and $\delta_{f}^{T}\left( \Phi\right)$ are the genetic trait modifiers at life sub-phase Φ for respectively wild and farm salmon. Note that in wild fish the frequency of wild alleles is 0.9, and thus $S^{\Phi}\approx0.9$ and$\delta^{T}\left( \Phi\right)\approx\delta_{w}^{T}\left( \Phi\right)$, where $\delta_{w}^{T}\left( \Phi\right)$ is the standard genetic value for wild salmon. Likewise, in farm fish the frequency of farm alleles is 0.1, and $S^{\Phi}\approx0.1$ and$\delta^{T}\left( \Phi\right)\approx\delta_{f}^{T}\left( \Phi\right)$, where $\delta_{f}^{T}\left( \Phi\right)$ is the standard genetic value for farm salmon. In hybrid salmon, $\delta_{f}^{T}\left( \Phi\right)\leq\delta^{T}\left( \Phi\right)\leq\delta_{w}^{T}\left( \Phi\right)$. Fig. B shows the linear mapping from allelic to genotypic value.

Table A summarises the notation for the genetic component of the IBSEM model.

| **Variable** | **Description** |
| --- | --- |
| $g\left( \Phi.j,l \right)$ | Allele value for locus *l* of *j^th^* copy of gene set $\Phi$ |
| $S^{\Phi}$ | Sum of genetic effects (life sub-phase / age *Φ*) |
| Γ | Parameter defining steepness of the exponential distribution of genetic effects |
| *Φ* | Life sub-phase / age (*eg,al,p0,p1,p2,sm,0SW,1SW,2SW,3SW,*) |
| $\delta^{T}\left( \Phi\right)$ | Genetic trait modifier at life sub-phase / age $\Phi$ |
| $\delta_{w}^{T}\left( \Phi\right)$ | Genetic trait modifier at life sub-phase / age $\Phi$ for wild salmon |
| $\delta_{f}^{T}\left( \Phi\right)$ | Genetic trait modifier at life sub-phase / age $\Phi$ for farm salmon |
| $\epsilon_{l}$ | Genetic effect of locus *l* |
| $\Phi$ | Life phase (*E*, *J*, *A*) |

**Table A** Genetic-model notation

**Fig A.** Assumed relative genetic effect for each of the 21 loci (L=21) on each phenotypic trait in the model. Note that locus 21 is neutral and has therefore no influence on the phenotype


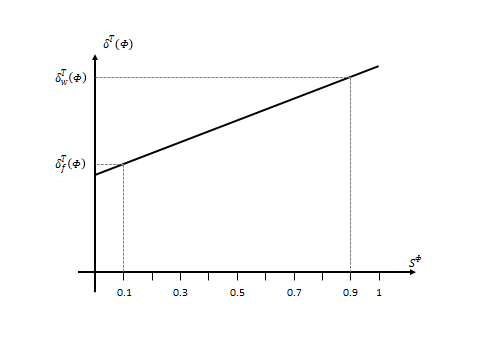


**Fig B.** Mapping from the allelic value $S^{\Phi}$ to genotypic value$\delta^{T}\left( \Phi\right)$ in traits.

A.4 DEMOGRAPHY

Individual salmon are subject to growth and mortality at all life phases. Smolting marks the transition from the freshwater to the oceanic phase. Sexual maturation happens at the end of the oceanic phase, when adults return to the river to reproduce. Precocious male parr may also mature during the freshwater phase, and take part to the reproduction process.

*A.4.1 Growth*

Somatic growth depends on individual variables such as the genotype, life phase, and current size of the salmon, as well as on environmental variables such as season, water temperature, and fish density. Finally, a random component is used to model environmental and developmental noise.

In the embryonic phase, mean alevin fork length $\lambda_{al}$ is determined by egg diameter $\lambda_{egg}$ according to the following linear relationship (Gilbey and Verspoor, 2005):

$\lambda_{al}=s_{1}\cdot l_{egg}+s_{2}$ (A.6)

where $s_{1}$ and $s_{2}$ are system parameters. The final alevin length $l_{al}$ is sampled from a Gaussian distribution centred on$\lambda_{al}$

$l_{al}$ = gaussian($\lambda_{al}$ mean, (${\sigma\left( al \right)\cdot\lambda}_{al}$ ) stdev) (A.7)

where the standard deviation ${\sigma\left( al \right)\cdot\lambda}_{al}$ replicates the distribution of alevin length used by Gilbey and Verspoor (2005).

Parr grow during the warm season (March–October) and rest in winter. Growth at the parr sub-phase is influenced by genotype, age, water temperature, and population density. Eq. (A.8) is widely used in the literature (Elliott and Hurley, 1997; Forseth et al., 2001) to determine the final weight $\omega_{X}\left( t+\Delta t \right)$ of an individual of initial weight $\omega_{X}\left( t \right)$ at age *X*, growing during a time interval$\Delta t$:

$\omega_{X}\left( t+\Delta t \right)=\left( {\omega_{X}\left( t \right)}^{b\left( X \right)}+a\left( X \right)\cdot b\left( X \right)\cdot\Delta t\cdot\left( T \right)\cdot\frac{1}{100} \right)^{\frac{1}{b\left( X \right)}}$ (A.8)

where $b\left( X \right)$ is a system parameter representing the allometric relationship between growth rate and fish mass at life age *X*, and $\left( T \right)$ (%) is the standardised mass-specific growth rate. The genetic effect on growth is included in the parameter $a\left( X \right)$

$a\left( X \right)=A\left( X \right)\cdot\delta^{A}\left( X \right)$ (A.9)

where $a\left( X \right)$ is akin to the state of ‘activity’ described in Piou and Prévost (2012). The activity $a\left( X \right)$ is used to modulate the growth rate according to the genotype via $\delta^{A}\left( X \right)$ ($\delta^{A}\left( X \right)$ defined as in Eq. A.4), and age via the system parameter$A\left( X \right)$.

The rate $\left( T \right)$ in Eq. A.9 depends on the average temperature *T* during$\Delta t$

$\left( T \right)=max\left[ d\left( X \right)\cdot\left( T-T_{L}\left( X \right) \right)\cdot\left( 1-e^{g\left( X \right)\cdot\left( T-T_{U}\left( X \right) \right)} \right),0 \right]$ (A.10)

where $d\left( X \right)$ and $g\left( X \right)$ are system parameters modelling the growth speed according to temperature at age *X*, and $T_{U}\left( X \right)$ and $T_{L}\left( X \right)$ are respectively the upper and lower critical water temperatures for growth at life sub-phase *X*. Note that for growth to take place, $T_{L}(X)\leq T\leq T_{U}(X)$.

The estimation of density effects ${dEff}_{g}(X)$ ($X\in\{p0, p1, p2\}$) on parr growth takes into account the effective density *ED* (Eq. 13, Post et al., 1999) of the population:

${dEff}_{g}\left( X \right)=\frac{1}{1+\beta_{dens}\left( X \right)\cdot ED}$ (A.11)

$ED=\sum_{i=1}^{N} \frac{\left( \frac{l_{i}\left( t \right)}{1000} \right)^{2}}{S}$ (A.12)

where $l_{i}\left( t \right)$ is the fork length of individual *i* at age *X* and time *t*, *S* is the river area, and $\beta_{dens}\left( X \right)$ is an age-dependent system parameter. Overall, parr growth is calculated as in Piou and Prévost (2012) including the density effects (Eq. A.12) in Eq. A.9:

$\omega_{X}\left( t+\Delta t \right)=\left( {\omega_{P}\left( t \right)}^{b(x)}+a\left( X \right)\cdot b\left( X \right)\cdot\Delta t\cdot\left( T \right)\cdot{dEff}_{g}\left( X \right)\cdot\frac{1}{100} \right)^{\frac{1}{b\left( X \right)}}$ (A.13)

where $X\in\{p0, p1, p2\}$ is the parr age group.

Density effects are considered not significant on growth in the smolt and adult phases, and $\omega_{X}\left( t+\Delta t \right)$ is calculated using Eq. (A.8). In both phases individuals grow all year, and growth depends on the activity state (Eq. A.9) which compounds age and genetic factors.

In order to take into account environmental stochasticity, the final weight of fish at the freshwater and adult phases is sampled from a Gaussian distribution centred in $\omega_{X}\left( t+\Delta t \right)$

$w_{X}\left( t+1 \right)$ = gaussian($\omega_{X}\left( t+1 \right)$ mean, $\sigma\left( X \right)\cdot\omega_{X}\left( t+1 \right)\mathrm{stdev}$) (A.14)

where $\sigma\left( X \right) (X\in\left\{ p0, p1, p2, sm, 0SW, 1SW, 2SW, 3SW \right\})$ is a sub-phase- / age-dependent system parameter. Fish weight and fork length are related according to Gilbey’s and Verspoor’s (2005) relationship:

$weight=\left( \frac{fork\_length}{42.855} \right)^{2.987}$ (A.15)

Table B summarises the notation for the growth equations of the IBSEM model.

| **Variable** | **Description** |
| --- | --- |
| $A\left( X \right)$ | Age-related effect on activity at age / life sub-phase *X* |
| $a\left( X \right)$ | State of activity at age / life sub-phase *X* |
| $b\left( X \right)$ | Allometric relationship between growth rate and fish mass at age / life sub-phase *X* |
| $d\left( X \right)$ | Growth speed according to temperature at age / life sub-phase *X* |
| ${dEff}_{g}\left( X \right)$ | Effect of population density on growth at parr age *X* |
| *ED* | Fish effective density |
| *g*$\left( X \right)$ | Temperature-related growth speed parameter at age / life sub-phase *X* |
| $l_{i}\left( t \right)$ | Fork length of individual *i* at time *t* |
| $l_{al}$ | Alevin fork length |
| $l_{egg}$ | Egg diameter |
| $s_{1}$ | Egg-alevin size relationship slope |
| $s_{2}$ | Egg-alevin size relationship offset |
| T | Temperature |
| $T_{L}\left( X \right)$ | Lower critical water temperature for growth at age / life sub-phase *X* |
| $T_{U}\left( X \right)$ | Higher critical water temperature for growth at age / life sub-phase *X* |
| $w_{X}\left( t \right)$ | Final weight at life sub-phase *X* at time *t* |
| *X* | Age / life sub-phase (*p0*, *p1*, *p2*, *sm*, 0*SW*, 1*SW*, 2*SW*, 3*SW*) |
| $\beta_{dens}(X)$ | Parameter modulating effect of effective fish density on growth at parr age *X* |
| $\Delta t$ | Time period |
| $\delta^{A}\left( X \right)$ | Genetic modifier on activity for life sub-phase $X$ |
| $\lambda_{al}$ | Mean alevin fork length |
| $\left( T \right)$ | Standardised mass-specific growth rate |
| $\sigma\left( X \right)$ | Standard deviation of weight spread (fraction of mean body weight) at age / life sub-phase *X* |
| $\omega_{X}\left( t \right)$ | Mean weight at age / life sub-phase *X* at time *t* |

**Table B** Growth parameters and variables notation

*A.4.2 Mortality*

Mortality depends on environmental variables including season, fish density, randomness, and individual variables including life sub-phase, genotype, and size. Finally, a random component is used to model environmental and developmental noise.

Density is taken into account only in the freshwater stage. Its effect ${dEff}_{m}\left( eg \right)$ on mortality is calculated using the relationship:

${dEff}_{m}(X)=\left( 1-\frac{\alpha(X)}{e^{\beta(X)\cdot\delta\left( X \right)}} \right)$ (A.16)

where $X$ is the life sub-phase, $\alpha(X)$ and $\beta(X)$ are sub-phase-dependent pre-defined parameters, and $\delta\left( X \right)$ is the density. In the embryonic phase, the density is calculated as egg/m^2^, in the parr sub-phase it corresponds to the effective density ED (Eq. A.12).

In the embryonic phase, egg-to-alevin mortality depends on genotype (wild has higher survival probabilities than farmed salmon), egg size, density, and randomness. Mortality increases exponentially with density according to Eq. (A.16) (Gilbey and Verspoor, 2005), and decreases linearly with egg size (Einum and Fleming, 2000; Skaala et al. 2012):

$\theta^{0}\left( eg \right)={m\cdot\omega}_{eg}+q$ (A.17)

where $\theta^{0}\left( eg \right)$ is the size-dependent component of egg-to-alevin mortality probability, $\omega_{eg}$ is the egg weight, and $m$ and $q$ are system parameters. Eqs. A.16 and A.17 are compounded in Eq. (A.18) to express the egg-to-alevin mortality probability $\theta\left( eg \right)$ for wild fish.

$\theta\left( eg \right)=\theta^{0}\left( eg \right)\cdot{dEff}_{m}(eg)$ (A.18)

The overall egg-to-alevin mortality probability (A.19) takes into account the genotype-dependent modifier$\delta^{s}\left( eg \right)$, which is calculated as in Eq. (A.4).

$m\left( eg \right)=1-\left( 1-\theta\left( eg \right) \right)\cdot\delta^{s}\left( eg \right)$ (A.19)

For each individual, a random number *r* in the interval $\left[ 0,1 \right]$ is drawn, and if *r*<$m\left( eg \right)$ the individual dies.

Parr mortality $\theta\left( X,s \right)$ depends on age and season via the variable$\theta^{0}\left( X,s \right)$:

$\theta^{0}\left( X,s \right)=1-\left( dsp\left( X,s \right) \right)^{\Delta t}\cdot$ (A.20)

where $dsp\left( X,s \right)$ is the daily survival probability (Piou and Prévost, 2012), $X\in\{p0, p1, p2\}$ is the parr age group, $s\in\{growth, rest\}$ is the season, and $\Delta t$ is the period in days. Density effects ${dEff}_{m}(X)$ are calculated according to Eq. (A.16), and accounted for in the calculation of density-dependent mortality $\theta\left( X,s \right)$:

$\theta\left( X,s \right)=\theta^{0}\left( X,s \right)\cdot{dEff}_{m}(X)$ (A.21)

The overall parr mortality probability $m\left( X,s \right)$ takes into account the origin of the fish by weighting the density-dependent survival probability $1-\theta\left( X,s \right)$ by the genotype-dependent modifier$\delta^{s}\left( X \right)$, which is calculated as in Eq. (A.4).

$m\left( X,s \right)=1-\left( 1-\theta\left( X,s \right) \right)\cdot\delta^{s}\left( X \right)$ (A.22)

For the smolt sub-phase, density effects on survival are considered negligible. The overall smolt mortality probability $m\left( sm,s \right)$ is computed taking into account only age ($\theta^{0}\left( X,s \right)$) and genetic $\delta^{s}\left( sm \right)$ factors:

$m\left( sm,s \right)=1-\left( 1-\theta^{0}\left( sm,s \right) \right)\cdot\delta^{s}\left( sm \right)$ (A.23)

Marine survival probability is related to individual size, genotype, and a random function. The overall mortality probability takes into account the origin of the fish by weighting adult survival probability $S$ (Piou and Prévost (2012) by the genotype-dependent modifier $\delta^{s}\left( A \right)$ (Eq. A.4):

$m\left( X \right)=1-S\cdot\delta^{s}\left( X \right)$ (A.24)

The adult survival probability $S$ takes into account the length $l_{A}$ of the individual and the duration of the period$\Delta t$:

${S=\left( 1-k_{1}\cdot{l_{A}}^{k_{2}} \right)}^{\frac{\Delta t}{30}}$ (A.25)

where $k_{1}$ and $k_{2}$ are pre-set parameters. Table C summarises the notation for the mortality equations of the IBSEM model.

| **Variable** | **Description** |
| --- | --- |
| ${dEff}_{m}(X)$ | Effect of density on mortality probability at age / life sub-phase *X* |
| $dsp\left( X,s \right)$ | Daily survival probability at age / life sub-phase *X* for season *s* |
| $k_{1}$ | Parameter affecting adult survival probability |
| $k_{2}$ | Parameter affecting adult survival probability |
| $l_{A}$ | Length of salmon (adult phase). |
| *m* | Slope of egg size effect on mortality |
| $m\left( X \right)$ | Overall mortality probability at adult age *X* |
| $m\left( eg \right)$ | Overall egg-to-alevin mortality probability |
| $m\left( X,s \right)$ | Overall juvenile mortality probability at age / life sub-phase *X* for season *s* |
| $q$ | Offset of egg size effect on mortality |
| *S* | Adult survival probability for wild fish |
| $w_{eg}$ | Egg weight |
| *X* | Age / life sub-phase (*eg*,*p0*, *p1*, *p2*, *sm*, 0*SW*, 1*SW*, 2*SW*, 3*SW*) |
| $\alpha(X)$ | Density-related parameter affecting mortality probability at age / life sub-phase *X* |
| $\beta(X)$ | Density-related parameter affecting mortality probability at age / life sub-phase *X* |
| $\Delta t$ | Time period |
| $\delta\left( X \right)$ | X=egg density (egg/m^2^), otherwise effective density ED |
| $\theta^{0}(eg)$ | Density-independent component of egg-to-alevin mortality probability |
| $\theta(eg)$ | Egg-to-alevin mortality probability for wild fish |
| $\theta^{0}\left( X,s \right)$ | Density-independent component of juvenile mortality probability at age / sub-phase *X* for season *s* |
| $\theta\left( X,s \right)$ | Wild parr mortality probability at age *X* for season *s* |
| $\delta^{s}\left( X \right)$ | Genetic modifier on survival at age / life sub-phase *X* |

**Table C** Mortality parameters and variables notation

*A.4.3 Maturation*

In the model, adult salmon spend between one and three winters at sea before they return to spawn. Maturation is related to genotype and randomness, with wild fish more likely to return earlier to spawn than the offspring of farmed salmon. At each sea age, individuals are associated to a fixed, age-dependent maturation probability$p\left( X \right)$:

$p\left( X \right)=P\left( X \right)\cdot\delta^{m}\left( X \right)$ (A.26)

where $P\left( X \right)$ ($X\in\left\{ 1SW, 2SW, 3SW \right\}$) is the age-related component of the adult maturation probability, and $\delta^{m}\left( X \right)$ is a genotype-dependent modifier $\delta^{s}\left( A \right)$ calculated as in Eq. (A.4). For each individual, a random number is drawn, and if $r<p\left( X \right)$ the individual matures.

Male individuals can also mature in freshwater as precocious parr. The male parr maturation probability(*p*) is a sigmoidal function of the length $l_{X}$ of the individual (Gilbey and Verspoor, 2005):

$p=\frac{Z}{1+Z}$ (A.27)

where

$Z=e^{K_{1}\cdot\left( l_{X}-K_{2} \right)}$ (A.28)

and $K_{1}$ and $K_{2}$ are pre-fixed parameters.

Based on empirical expertise at the IMR, we gave male parr that matured the year before and didn’t migrate to sea, a parr maturation probability two times larger than the probability of juveniles that had never matured before. The growth season ($\Delta t$, Eq. A.8) of maturing parr is reduced one month (30 days) to reflect that they stop growing as resources are employed for gonad development.

The notation concerning the maturation process is given in Table D.

*A.4.4 Smoltification*

In the model, the probability $p$ of smolting the following May is calculated at the end of October for each individual using the following logistic function

$p=\left\{ \begin{matrix} l_{X}\geq\tau\to\frac{Z}{1+Z} \\ 0 \end{matrix} \right.$ (A.29)

where $\tau$ is the length threshold for smolting, and $Z$ is calculated as in Eq. (A.28). Eq. (29) is akin to Eq. (27), with the addition of a of a minimal length threshold$\tau$.

The notation concerning the smoltification process is given in Table D.

| **Variable** | **Description** |
| --- | --- |
| $K_{1}$ | Parameter determining the steepness of the parr maturation/smolting probability curve (mm^-1^) |
| $K_{2}$ | Fork length of parr having maturation/smolting probability $\mu=1/2$ (mm) |
| $l_{X}$ | Length of parr at age *X*. (mm) |
| $P\left( X \right)$ | Age-related component of adult maturation probability |
| $p$ | Parr maturation/smolting probability |
| $p\left( X \right)$ | Adult maturation probability |
| *X* | Sea age (0SW, 1SW, 2SW, 3SW) |
| $\delta^{m}\left( X \right)$ | Genetic modifier on adult maturation probability at age *X* (1SW, 2SW, 3SW) |
| $\mu\left( X \right)$ | Adult maturation probability at sea age *I* |
| $\tau$ | October length threshold on smolting |

**Table D** Maturation and smolting parameters and variables notation

*A.4.5 Reproduction*

The pool of reproducing individuals is composed of sexually mature male parr, adult males, and adult females.

Farmed escapees are given a lower spawning success than fish of any genetic make-up that are born in the wild (Fleming et al., 1996). The reduced spawning success of farm individuals is quantified as a fraction *rs* of the success of wild born fish.

Male parr undergo a pre-spawning mortality mimicking deaths due to attacks by large adult males on the spawning sites. Every year, the parr pre-spawning mortality probability $m_{pre}$ is randomly sampled with uniform probability from a pre-set range of values

$m_{pre}=uniform\left( \left[ m_{min},m_{max} \right] \right)$ (A.30)

where $m_{min}$ and $m_{max}$ are fixed system parameters.

Adult male and female salmon undergo post-spawning mortality with fixed probability$m_{post}$.

The mean number of eggs $\nu_{eggs}$ spawned by a female salmon and their average weight $\omega_{egg}$ depend on the spawner’s body mass $\boldsymbol{w}_{\boldsymbol{A}}\left( \boldsymbol{F} \right)$ (Jonsson et al., 1996; Piou and Prévost, 2012).

$\boldsymbol{\nu}_{\boldsymbol{eggs}}\boldsymbol{=}\boldsymbol{e}^{\boldsymbol{c}_{\boldsymbol{1}}\boldsymbol{\cdot ln}\left[ \boldsymbol{w}_{\boldsymbol{A}}\left( \boldsymbol{F} \right) \right]\boldsymbol{+}\boldsymbol{c}_{\boldsymbol{2}}}$ (A.31)

$\boldsymbol{\omega}_{\boldsymbol{egg}}\boldsymbol{=}\frac{\boldsymbol{e}^{\boldsymbol{c}_{\boldsymbol{3}}\boldsymbol{\cdot ln}\left( \boldsymbol{w}_{\boldsymbol{A}}\left( \boldsymbol{F} \right) \right)\boldsymbol{+}\boldsymbol{c}_{\boldsymbol{4}}}}{\boldsymbol{10}}\boldsymbol{\cdot}\frac{\boldsymbol{1}}{\boldsymbol{1000}}$ (A.32)

where $c_{1}$, $c_{2}$, $c_{3}$, and $c_{4}$ are system parameters. The final number $n_{eggs}$ and individual weight $w_{egg}$ of the eggs is sampled from Gaussian distribution functions.

$n_{eggs}$ = gaussian($\nu_{eggs}$ mean, $\sigma_{NE}\cdot\nu_{eggs}$ stdev) (A.33)

$w_{egg}$ = gaussian($\boldsymbol{\omega}_{\boldsymbol{egg}}$ mean, $\sigma_{WE}\cdot\boldsymbol{\omega}_{\boldsymbol{egg}}$ stdev) (A.34)

where $\sigma_{NE}$ and $\sigma_{NE}$ are the standard deviations (expressed as fraction of the average value) of the spreads of respectively the number of eggs per female, and the individual egg weight.

Egg weight and diameter are related using the relationship by Fleming and Ng (1987)

$weight=\left( \frac{diameter}{0.69} \right)^{-2.889}\cdot\frac{1}{1000}$ (A.35)

For each female, 7 males are involved in fertilisation (2 mature adults and 5 sexually mature parr, see for example Taggart, 2001). The percentage of eggs fertilised by sexually mature parr is determined randomly from a normal distribution defined by a mean of 30% and a standard deviation of 10% (as in Gilbey and Verspoor, 2005). The males are divided in two pools (adult and sexually mature parr). For each female, the mates are selected from the two pools based on the individual body mass.

$\boldsymbol{sp}_{\boldsymbol{i}}\boldsymbol{=}\frac{\boldsymbol{w}_{\boldsymbol{i}}\left( \boldsymbol{M} \right)}{\sum_{\boldsymbol{j=0}}^{\boldsymbol{M}} \boldsymbol{w}_{\boldsymbol{j}}\left( \boldsymbol{M} \right)}$ (A.36)

where ${sp}_{i}$ is the selection probability of male spawner *i*, and $w_{i}\left( M \right)$ is the weight of male spawner *i*.

Once a female has been mated, the proportion of eggs fertilised by sexually mature parr is drawn, and for each egg a male is randomly picked either from the precocious or the adult selected mates. The notation relative to the spawning process is given in Table E.

| **Variable** | **Description** |
| --- | --- |
| $c_{1}$ | System parameter for the determination of the number of the eggs spawned by a female |
| $c_{2}$ | System parameter for the determination of the number of the eggs spawned by a female |
| $c_{3}$ | System parameter for the determination of the weight of the eggs of a female |
| $c_{4}$ | System parameter for the determination of the weight of the eggs of a female |
| $m_{pre}$ | Pre-spawning juvenile mortality probability |
| $m_{min}$ | Juvenile minimum pre-spawning mortality probability |
| $m_{max}$ | Juvenile maximum pre-spawning mortality probability |
| $m_{post}$ | Adult post-spawning mortality probability |
| *rs*(*sex*) | Spawning success of farm escapees (*sex*=*male*, *female*) |
| ${sp}_{i}$ | Selection probability for adult/juvenile male spawner *j* |
| $w_{A}\left( X \right)$ | Weight of adult spawner (sex=*M*,*F*) |
| $w_{egg}$ | Weight of individual egg |
| $n_{egg}$ | Number of eggs spawned by a female salmon |
| $\nu_{egg}$ | Average number of eggs spawned by a female salmon |
| σ | fixed fraction of the returners that strays into/from neighbouring rivers |
| $\sigma_{NE}$ | Standard deviation of spread of number of eggs per female (fraction of average number) |
| $\sigma_{WE}$ | Standard deviation of spread of eggs weight (fraction of average number) |
| *χ* | Frequency of wild allele in the genotype of strayers |
| $\omega_{egg}$ | Average weight of eggs spawned by a female |

**Table E** Reproduction equations

*A.4.6 Straying*

Each year, a fixed fraction σ of the returners is considered to stray into neighbouring rivers (and thus do not return to the focal river). Within IBSEM, strayers are randomly selected and removed from the population, and each strayer is replaced with a newly generated individual.

The genotype of the immigrant strayer is randomly generated and composed mainly of wild type (‘1’) alleles. The frequency of the wild allele in the genotype of strayers is a pre-set parameter *χ*. To account for the lack of local adaptation to the river, the new individual is given a frequency *χ*=0.8 of wild alleles that is slightly lower that the frequency in wild salmon (0.9, see Section 2.3).

Some phenotypic attributes of the immigrant strayer need to be defined in order to determine the mating probabilities (males, Eq. A.36) or the number and size of eggs (females, Eq. A.31 and A.32). In the model the phenotypic attributes of the immigrant individual replicate those of the outgoing strayer.

The notation for the straying process is included in Table E.

LIST OF REFERENCES

Einum S, Fleming IA. Highly fecund mothers sacrifice offspring survival to maximize fitness. Nature 2000; 405(6786): 565-567.

Elliott JM, Hurley MA. A functional model for maximum growth of Atlantic Salmon parr, Salmo salar, from two populations in northwest England. Funct. Ecol. 1997; 11(5): 592-603.

Fleming IA, Ng S. Evaluation of techniques for fixing, preserving, and measuring salmon eggs. Can. J. Fish. Aquat. Sci. 1987; 44(11): 1957-1962.

Fleming IA, Jonsson B, Gross MR, Lamberg A. An experimental study of the reproductive behaviour and success of farmed and wild Atlantic salmon (Salmo salar). J. Appl. Ecol. 1996; 33(4): 893-905.

Forseth T, Hurley MA, Jensen AJ, Elliott JM. Functional models for growth and food consumption of Atlantic salmon parr, Salmo salar, from a Norwegian river. Freshw. Biol. 2001; 46: 173-186.

Gilbey J, Verspoor E. Simulation Modelling of the Genetic Interactions between Farmed and Wild Fish - Description, Parameterisation and Corroboration of Demographic Model Components. Report for Fisheries Research Services, Crown copyright, 2005.

Jonsson N, Jonsson B, Fleming IA. Does early growth cause a phenotypically plastic response in egg production of Atlantic salmon? Funct. Ecol. 1996; 10(1): 89-96.

Piou C, Prévost E. A demo-genetic individual-based model for Atlantic salmon populations: Model structure, parameterization and sensitivity. Ecol. Model. 2012; 231: 37-52.

Post JR, Parkinson EA, Johnston NT. Density-dependent processes in structured fish populations: interaction strengths in whole-lake experiments, Ecol. Monogr. 1999; 69(2): 155-175.

Skaala Ø, Glover KA, Barlaup BT, Svåsand T, Besnier F, Hansen MM, Borgstrøm R. Performance of farmed, hybrid, and wild Atlantic salmon (Salmo salar) families in a natural river environment. Can. J. Fish. Aquat. Sci. 2012; 69(12): 1994-2006.

Taggart JB, McLaren IS, Hay DW, Webb JH, Youngson AF. Spawning success in Atlantic salmon (Salmo salar L.): a long‐term DNA profiling‐based study conducted in a natural stream. Molecular Ecology, 2001; 10(4), 1047-1060.
