# Supplementary material for: IBSEM: An Individual-Based Atlantic Salmon Population Model
Source: PLoS One. 2015 Sep 18;10(9):e0138444. doi: 10.1371/journal.pone.0138444 (PMC4575158; doi:10.1371/journal.pone.0138444)
Supplement: S2 File — (DOCX) [file pone.0138444.s002.docx]

**IBSEM: AN INDIVIDUAL-BASED ATLANTIC SALMON POPULATION MODEL**

Marco Castellani^1,2^, Mikko Heino^1,3,4^, John Gilbey^5^, Hitoshi Araki^6^, Terje Svåsand^1^, Kevin A. Glover^1^

^1^ *Institute of Marine Research, P.O. Box 1870, Nordnes, N-5817 Bergen, Norway*

^2^ School of Mechanical Engineering, University of Birmingham, Birmingham B15 2TT, UK.

^3^ *Department of Biology, University of Bergen, Bergen, Norway*

^4^ *IIASA, Laxenburg, Austria*

^5^ *Marine Scotland Science, Freshwater Laboratory, Faskally, Pitlochry, Scotland, U.K. PH16 5LB*

^6^ *Research Faculty of Agriculture, Hokkaido University, 060-8589, Japan*

# ONLINE S2 FILE

# PARAMETERIZATION OF MODEL

B.1 PHYSICAL ENVIRONMENT

The environmental conditions for the freshwater phase are modelled on those encountered in the Os river (Rådgivende Biologer, 2012). For the oceanic phase, the temperatures reproduce the monthly average sea surface temperatures (SSTs) measured in the 30 years period 1982-2012 in the Norwegian Sea (NOAA_ERSST_V3 data provided by the NOAA/OAR/ESRL PSD, Boulder, Colorado, USA, from their Web site at <http://www.esrl.noaa.gov/psd/>).

Table A gives the standard values of the environmental parameters.

| **Freshwater stage** | | **Oceanic stage** | |
| --- | --- | --- | --- |
| **Oselva river, Hordaland (N)** | | **Norwegian Sea** | |
| **River area=250,000 m^2^** | | **-** | |
| **Average water temperatures (^º^C)** | | | |
| Month | River | | Sea |
| January | 2.00 | | 4.82 |
| February | 2.00 | | 4.65 |
| March | 2.00 | | 4.67 |
| April | 5.00 | | 5.16 |
| May | 11.00 | | 6.3 |
| June | 16.00 | | 7.91 |
| July | 18.00 | | 9.15 |
| August | 18.00 | | 9.3 |
| September | 14.00 | | 8.26 |
| October | 9.00 | | 6.96 |
| November | 5.00 | | 5.89 |
| December | 3.00 | | 5.23 |
| Standard deviation $\sigma_{T}$ on monthly temperatures | 1.0 | | 0.3 |

**Table A** Environmental parameters.

B.2 DEMOGRAPHY

The Individual-Based Salmon Eco-genetic Model (IBSEM) reproduces the life cycle of an Atlantic salmon (Salmo salar L.) population. The model divides the life history of the individuals into three main phases: embryonic (egg to the end of endogenous feeding on its embryonic yolk-sac reserves, E), freshwater (juvenile, J), and oceanic (adult, A).

The equations described in S1 File have been parameterized to reproduce the demographics Atlantic salmon in the river Os in Norway. The following tables list the settings of the parameters.

*B.2.1 Growth*

| **Parameter** | **Phase** | ***X* (Age)** | | **Value** | **Measurement unit** | **Source** |
| --- | --- | --- | --- | --- | --- | --- |
| $A\left( X \right)$ | *J* | *p0* | | 0.75 | g/days | * |
|  |  | *p1* | | 0.7 | g/days | * |
|  |  | *p2* | | 0.5 | g/days | * |
|  |  | *sm* (young-of-the-year) | | 0.7 | g/days | * |
|  |  | *sm* (older) | | 0.6 | g/days | * |
|  | *A* | *0SW* | | 4.6 | g/days | * |
|  |  | *1SW*, *2SW*, *3SW* | | 2.2 | g/days | * |
| $b\left( X \right)$ | *J*, *A* | *p0*, *p1*, *p2*, *sm* | | 0.31 | - | (1) |
| $d\left( X \right)$ | *J* | *p0*, *p1*, *p2*, *sm* | | 0.374 | 1/^º^C | (1) |
|  | *A* | *0SW*, *1SW*, *2SW*, *3SW* | | 0.33 | 1/^º^C | * |
| *g*$\left( X \right)$ | *J* | *p0*, *p1*, *p2*, *sm* | | 0.201 | 1/^º^C | (1) |
|  | *A* | *0SW*, *1SW*, *2SW*, *3SW* | | 0.2 | 1/^º^C | * |
| $s_{1}$ | *E* | *eg* | | 1.6345 | - | (2) |
| $s_{2}$ | *E* | *eg* | | 12.991 | mm | (2) |
| $T_{L}\left( X \right)$ | *J* | *p0*, *p1*, *p2* | | 7 | ^º^C | (3) ** |
|  |  | *sm* | | 0 | ^º^C | (3) ** |
|  | *A* | *0SW*, *1SW*, *2SW*, *3SW* | | 2 | ^º^C | * |
| $T_{U}\left( X \right)$ | *J* | *p0*, *p1*, *p2* | | 24 | ^º^C | (3) ** |
|  |  | *sm* | | 24 | ^º^C | (3) ** |
|  | *A* | *0SW*, *1SW*, *2SW*, *3SW* | | 20 | ^º^C | * |
| $\beta_{dens}(X)$ | *J* | *p0* | | 20 | - | (4) ** |
|  |  | *p1*, *p2* | | 70 | - | (4) ** |
| $\delta_{f}^{A}\left( X \right)$ | *J* | *p0*, *p1*, *p2* | | 1.3 | - | * |
|  |  | *sm* | | 2.0 | - | * |
|  | *A* | *0SW*, *1SW*, *2SW*, *3SW* | | 1.2 | - | * |
| $\delta_{w}^{A}\left( X \right)$ | *all* | *all* | | 1 | - | * |
| $\sigma\left( X \right)$ | *E* | *al* | | 0.035 | - | (2) |
|  | *J* | *p0* | | 0.25 | - | * |
|  |  | *p1*, *p2* | | 0.08 | - | * |
|  |  | *sm* | | 0.09 | - | * |
|  | *A* | *0SW*, *1SW*, *2SW*, *3SW* | | 0.1 | - | * |
| 1. Elliott and Hurley, (1997) 2. Gilbey and Verspoor, (2005) 3. Forseth et al., (2001) 4. Piou and Prevost (2012) | | | * fitted to experimental data (Rådgivende Biologer, 2012)  ** modified to fit experimental data (Rådgivende Biologer, 2012) | | | |

**Table B** Growth parameters setting.

*B.2.2 Mortality*

| **Parameter** | **Phase** | ***X* (Age)** | | **Value** | **Measurement unit** | **Source** |
| --- | --- | --- | --- | --- | --- | --- |
| $dsp\left( X,s \right)$ | J | *p0*, *s*=*warm* | | 0.983 | - | (1) ** |
|  |  | *p0*, *s*=*cold* | | 0.9988 | - | (1) ** |
|  |  | *p1*, *p2*, *s*=*warm* | | 0.9986 | - | (1) ** |
|  |  | *p1*, *p2*, *s*=*cold* | | 0.999 | - | (1) ** |
|  |  | *sm* | | 0.999 | - | (1) ** |
| $k_{1}$ | A | *0SW*, *1SW*, *2SW*, *3SW* | | 950 | 1/mm | (1) ** |
| $k_{2}$ | A | *0SW*, *1SW*, *2SW*, *3SW* | | -1.55 | - | (1) ** |
| *m* | E | eg | | -3.16 | 1/g | * |
| q | E | *eg* | | 1.12 | - | * |
| $\alpha(X)$ | E | *eg* | | 0.62 | - | ** *** |
|  | J | *p0* | | 0.28 | - | ** *** |
|  |  | *p1*, *p2*, | | 0.3 | - | ** *** |
| $\beta(X)$ | E | *eg* | | 0.26 | m^2^/egg | ** *** |
|  | J | *p0* | | 830 | - | ** *** |
|  |  | *p1*, *p2*, | | 280 | - | ** *** |
| $\delta_{f}^{S}\left( X \right)$ | E | eg | | 0.8 | - | * |
|  | J | *p0, p1*, *p2*, *sm* | | 0.8 | - | * |
|  | A | *0SW*, *1SW*, *2SW*, *3SW* | | 0.6 | - | * |
| $\delta_{w}^{S}\left( X \right)$ | *all* | *all* | | 1 | - | * |
| 1. Piou and Prevost (2012) 2. Gilbey and Verspoor, (2005) 3. Forseth et al., (2001) | | | * fitted to experimental data (Rådgivende Biologer, 2012)  ** fitted to exponential curve in Gilbey and Verspoor, (2005)  *** modified to fit experimental data (Rådgivende Biologer, 2012) | | | |

**Table C** Mortality parameters setting.

*B.2.3 Maturation and Smolting*

| **Maturation** | | | | |  |  |
| --- | --- | --- | --- | --- | --- | --- |
| **Parameter** | **Phase** | ***X* (Age)** | **Value** | | **Measurement unit** | **Source** |
| $K_{1}$ | J | *p0*, *p1 p2* | 0.13 | | 1/mm | (1) ** |
| $K_{2}$ | J | *p0*, *p1 p2* | 107 | | mm | (1) ** |
| $P\left( X \right)$ | A | *0SW* | 0 | | - | (3) |
|  |  | *1SW* | 0.4 | | - | (3) ** |
|  |  | *2SW* | 0.85 | | - | (3) ** |
|  |  | *3SW* | 1 | | - | (3) ** |
| $\delta_{f}^{m}\left( X \right)$ | A | *0SW* | 0 | | - | * |
|  |  | *1SW* | 0.25 | | - | * |
|  |  | *2SW* | 0.47 | | - | * |
|  |  | *3SW* | 1 | | - | * |
| $\delta_{w}^{m}\left( X \right)$ | *all* | *all* | 1 | | - | * |
| **Smolting** | | | | |  |  |
| **Parameter** | **Phase** | ***X* (Age)** | **Value** | |  |  |
| $K_{1}$ | J | *p0*, *p1 p2* | 0.2 | | 1/mm | (2) (3) ** |
| $K_{2}$ | J | *p0*, *p1 p2* | 103 | | mm | (2) (3) ** |
| $\tau$ | J | *p0*, *p1 p2* | 90 | | mm | * |
| 1. Gilbey and Verspoor, (2005) 2. Piou and Prevost (2012) 3. Hedger et al. (2013) | | | | * fitted to experimental data (Rådgivende Biologer, 2012)  ** modified to fit experimental data (Rådgivende Biologer, 2012) | | |

**Table D** Maturation and smolting parameters setting.


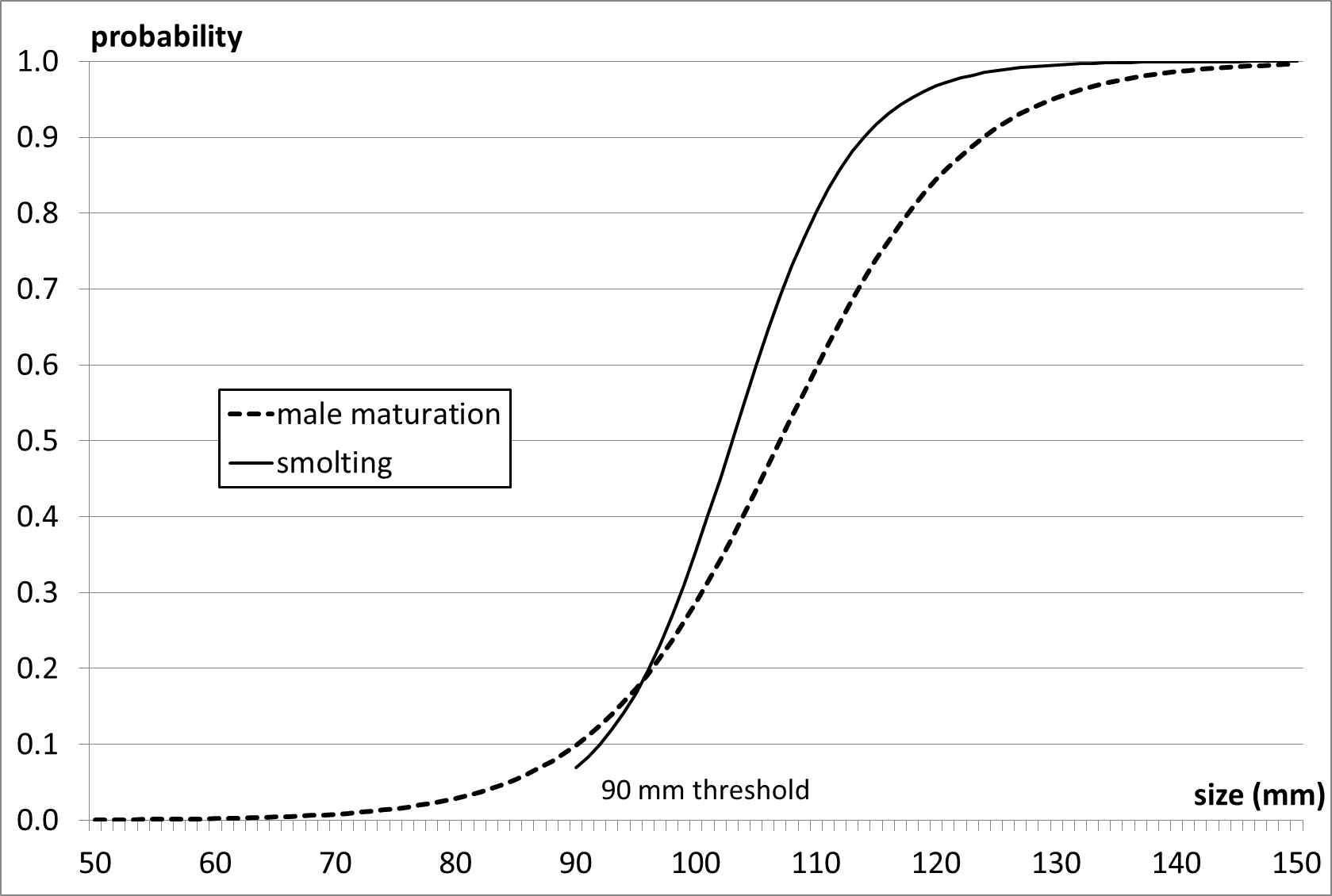


**Fig A.** Fork length-dependent male parr maturation and smolting probabilities. Equations and parameters as described in Tables D in S1 File and D.

*B.2.4 Reproduction and Straying*

| **Parameter** | **Phase** | ***X* (Age)** | | **Value** | **Measurement unit** | **Source** |
| --- | --- | --- | --- | --- | --- | --- |
| $c_{1}$ | A | *1SW*, *2SW*, *3SW* | | 0.86 | 1/g | (1) |
| $c_{2}$ | A | *1SW*, *2SW*, *3SW* | | 1.63 | - | (1) |
| $c_{3}$ | A | *1SW*, *2SW*, *3SW* | | 0.166 | 1/g | (1) |
| $c_{4}$ | A | *1SW*, *2SW*, *3SW* | | 5.68 | - | (1) |
| $m_{min}$ | J | *p0*, *p1 p2* | | 0.1 | - | * |
| $m_{max}$ | J | *p0*, *p1 p2* | | 0.3 | - | * |
| $m_{post}$ | A | *1SW*, *2SW*, *3SW* | | 0.95 | - | * |
| *rs*(*sex*) | A | *1SW*, *2SW*, *3SW* | | *sex=male 0.05 sex=female 0.3* | - | (2) |
| σ | A | *1SW*, *2SW*, *3SW* | | 0.05 | - | * |
| $\sigma_{NE}$ | A | *1SW*, *2SW*, *3SW* | | 0.1 | - | * |
| $\sigma_{WE}$ | A | *1SW*, *2SW*, *3SW* | | 0.02 | - | * |
| *χ* | A | *1SW*, *2SW*, *3SW* | | 0.8 | - | *** |
| 1. Jonsson et al., (1996) 2. Fleming et al., (1996) | | | * fitted to experimental data (Rådgivende Biologer, 2012)  ** modified to fit experimental data (Rådgivende Biologer, 2012)  *** heuristically set | | | |

**Table E** Reproduction and strayers parameters setting.

B.3 SOFTWARE IMPLEMENTATION

The IBSEM model is implemented in C++. With the above parameters, the overall running time (simulating 200 years of evolution) takes approximately 43 minutes on an Intel Core i7-3610QM processor of speed 2.30GHz and memory 32.0 GB, using Windows 7 64-bit operating system.

B.3 SENSITIVITY TESTS

| **Parameter** | **Phase** | ***X* (Age)** | **Standard value**  **(Scenarios 1, 2, 3)** | **Scenario 4** | **Scenario 5** |
| --- | --- | --- | --- | --- | --- |
| $\delta_{f}^{A}\left( X \right)$ | *J* | *p0*, *p1*, *p2* | 1.3 | 1.15 | 1.45 |
|  |  | *sm* | 2.0 | 1.5 | 2.5 |
|  | *A* | *0SW*, *1SW*, *2SW*, *3SW* | 1.2 | 1.1 | 1.3 |
| $\delta_{f}^{S}\left( X \right)$ | E | eg | 0.8 | 0.9 | 0.7 |
|  | J | *p0, p1*, *p2*, *sm* | 0.8 | 0.9 | 0.7 |
|  | A | *0SW*, *1SW*, *2SW*, *3SW* | 0.6 | 0.8 | 0.4 |

**Table F** Sensitivity tests.

**References**

Elliott JM, Hurley MA. A functional model for maximum growth of Atlantic Salmon parr, Salmo salar, from two populations in northwest England. Funct. Ecol. 1997; 11(5): 592-603.

Fleming IA, Jonsson B, Gross MR, Lamberg A. An experimental study of the reproductive behaviour and success of farmed and wild Atlantic salmon (Salmo salar). J. Appl. Ecol. 1996; 33(4): 893-905.

Forseth T, Hurley MA, Jensen AJ, Elliott JM. Functional models for growth and food consumption of Atlantic salmon parr, Salmo salar, from a Norwegian river. Freshw. Biol. 2001; 46: 173-186.

Gilbey J, Verspoor E. Simulation Modelling of the Genetic Interactions between Farmed and Wild Fish - Description, Parameterization and Corroboration of Demographic Model Components. Report for Fisheries Research Services, Crown copyright, 2005.

Hedger RD, Sundt-Hansen LE, Forseth T, Diserud OH, Ugedal O, Finstad AG. Modelling the complete life-cycle of Atlantic salmon (Salmo salar L.) using a spatially explicit individual-based approach. Ecol. Model. 2013; 248: 119-129.

Jonsson N, Jonsson B, Fleming IA. Does early growth cause a phenotypically plastic response in egg production of Atlantic salmon? Funct. Ecol. 1996; 10(1): 89-96.

Piou C, Prévost E. A demo-genetic individual-based model for Atlantic salmon populations: Model structure, parameterization and sensitivity. Ecol. Model. 2012; 231: 37-52.

Rådgivende Biologer. Fiskeundersøkingar i Os i Hordaland i 2010 og 2011. Bestandsutvikling 1991-2010. Technical report AS 1527, <http://www.radgivende-biologer.no/uploads/Rapporter/1527.pdf>, 2012, accessed June 2015.
