# Supplementary material for: IBSEM: An Individual-Based Atlantic Salmon Population Model
Source: PLoS One. 2015 Sep 18;10(9):e0138444. doi: 10.1371/journal.pone.0138444 (PMC4575158; doi:10.1371/journal.pone.0138444)
Supplement: S1 Minimal dataset — (ZIP) [file pone.0138444.s003.zip › read me.docx]

This zipped file contains all data generated by the IBSEM model. It contains all values behind statistics, graphs, figures, tables, presented in the manuscript.
